# Wearable Technology and Analytics as a Complementary Toolkit to Optimize Workload and to Reduce Injury Burden

**DOI:** 10.3389/fspor.2020.630576

**Published:** 2021-01-21

**Authors:** Dhruv R. Seshadri, Mitchell L. Thom, Ethan R. Harlow, Tim J. Gabbett, Benjamin J. Geletka, Jeffrey J. Hsu, Colin K. Drummond, Dermot M. Phelan, James E. Voos

**Affiliations:** ^1^Department of Biomedical Engineering, Case Western Reserve University, Cleveland, OH, United States; ^2^Case Western Reserve University School of Medicine, Cleveland, OH, United States; ^3^Department of Orthopaedic Surgery, University Hospitals Cleveland Medical Center, Cleveland, OH, United States; ^4^Sports Medicine Institute, University Hospitals Cleveland Medical Center, Cleveland, OH, United States; ^5^Gabbett Performance Solutions, Brisbane, QLD, Australia; ^6^Centre for Health Research, University of Southern Queensland, Ipswich, QLD, Australia; ^7^Division of Cardiology, David Geffen School of Medicine, University of California, Los Angeles, Los Angeles, CA, United States; ^8^Sports Cardiology, Hypertrophic Cardiomyopathy Program, Sanger Heart and Vascular Institute, Atrium Health, Charlotte, NC, United States

**Keywords:** wearable sensors, artificial intelligence, machine learning, sports medicine, return-to-play, sports cardiology, workload optimization

## Abstract

Wearable sensors enable the real-time and non-invasive monitoring of biomechanical, physiological, or biochemical parameters pertinent to the performance of athletes. Sports medicine researchers compile datasets involving a multitude of parameters that can often be time consuming to analyze in order to create value in an expeditious and accurate manner. Machine learning and artificial intelligence models may aid in the clinical decision-making process for sports scientists, team physicians, and athletic trainers in translating the data acquired from wearable sensors to accurately and efficiently make decisions regarding the health, safety, and performance of athletes. This narrative review discusses the application of commercial sensors utilized by sports teams today and the emergence of descriptive analytics to monitor the internal and external workload, hydration status, sleep, cardiovascular health, and return-to-sport status of athletes. This review is written for those who are interested in the application of wearable sensor data and data science to enhance performance and reduce injury burden in athletes of all ages.

## Introduction

Sports scientists continually seek newer technologies, data platforms, and therapies to help athletes perform at their highest level while reducing the risk of injury over an arduous season. Sports teams have recently utilized wearable sensors such as the Catapult OptimEye S5 (Wellman et al., 2016; Li et al., 2020), Zebra RFID tag (Zebra and The NFL, 2019), and Zephyr BioHarness (Nazari et al., 2018) to provide a quantifiable measure of the exertional output of the athlete, described as “workload.” The collection of such data has allowed some professional teams to show a relationship between workload and injury rates (Li et al., 2020). The creation of open-source repositories of wearable data will facilitate collaborations between academics and sports teams to develop injury assessment and workload models to maximize health and performance (Vamathevan et al., 2019). There remains a need to better understand how biomechanical, physiological, and biochemical data relates to injury risk and what technology and predictive models can measure and translate the data into clinically-relevant assessments. The technology does exist, but its understanding and use in injury-prevention has not been extensively studied in the sports medicine community.

The application of machine learning (ML) has emerged in sports such as in Major League Baseball (MLB); where publicly available tools such as Statcast track player performance data and provides insight into future production based on changes in batting average, on base percentage, runs batted in (RBIs), and other performance-related metrics. Furthermore, in the National Basketball Association (NBA), teams have utilized ML models to increase season ticket retention rates between seasons (Goh, 2019). The application of these models in sports medicine remains limited, prompting further research (Claudino et al., 2019). Predictive models have the potential to act as an automated data analyst capable of providing insight into the athlete's condition. The performance of predictive models in the literature thus far has been poor due, in part, to their small sample sizes with low injury rates, but also because our incomplete understanding of the determinants of injury and how these variables behave in a dynamic system of athlete performance (Seow et al., 2020).

**Figure 1:**
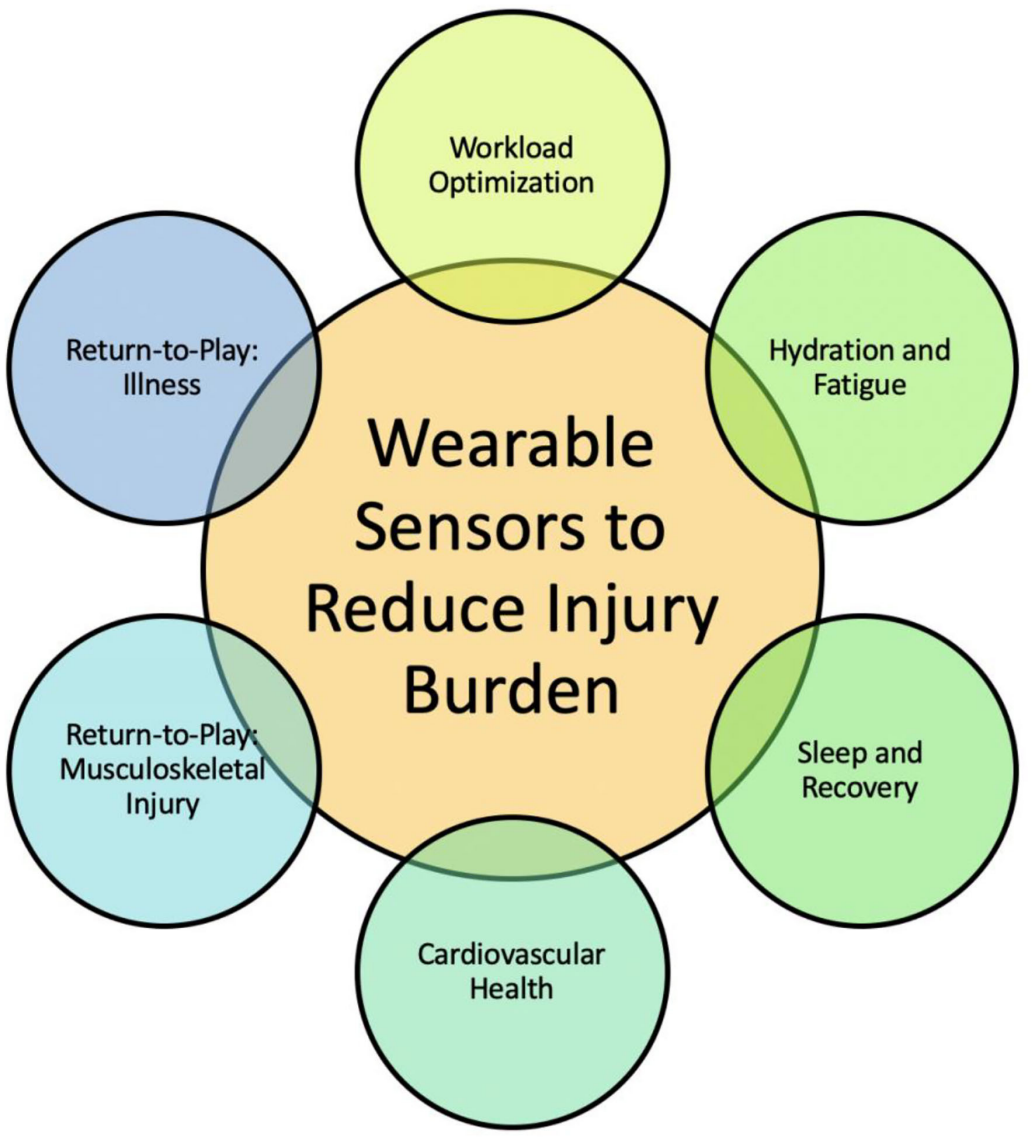
Goal of this review is to provide opportunities for engineers, clinicians, and data scientists to leverage advances in each of the six thrusts toward reducing injury burden. The integration of accurate data from wearable sensor technology into predictive models toward monitoring athlete safety and performance is a needed and growing field of study.

This review discusses our understanding of how wearable technology and analytics are being used to measure select biomechanical and physiologic parameters in the athlete and the predictive modeling techniques described in the literature to reduce injury burden (Figure 1). In addition, given the unique circumstances of the COVID-19 pandemic, this review discusses a possible role of wearable technology in guiding the training of athletes as they return from the COVID-19 lockdown. Definitions of key terms presented in this review are introduced (Table 1).

**Table 1:** Definition of key terms presented or indirectly highlighted in this review as it pertains to the use of predictive analytics and wearable sensors to reduce injury burden.

**Key terms**	**Definition**
Acute workload	Workload of an athlete over a short duration (typically one-session to 1-week). This value contains both training- and match-load information over the set time period and is analogous to “fatigue.”
Area under the curve	Measure of how well a parameter or model can distinguish between two diagnostic groups (injured/healthy).
Biomechanical sensor	Sensor which measures the movement (e.g., distance traveled, velocity, and acceleration) or forces exerted on the musculoskeletal system (e.g., force on tibia, foot, ACL).
Biochemical sensor	Sensor which measures biomarkers from bodily fluids such as eccrine or apocrine sweat.
Chronic workload	Workload of an athlete over a 3-weeks or longer duration. Chronic workload provides a clear indication of what an athlete has done leading up to the present training or match day and provides an indication of an athlete's capacity (also analogous to an athlete's “fitness” level).
Diagnostic tests	Protocol a physician follows to diagnose an ailment (e.g., Lachman or Pivot-Shift tests performed by an orthopedic surgeon before swelling and effusion sets in to aid in the diagnosis of a torn ACL to triage which athletes need MRI).
Acute-to-chronic workload ratio	Ratio of the acute workload to the chronic workload. The ratio provides an indication of the training an athlete has performed relative to their capacity. This data can then be fed into models to assess the likelihood the athlete may suffer an injury manifesting from repeated-overuse (or underuse).
External workload	Measure of the physical work done during training and/or competition.
Gold standard	Standard of care treatment (current and preferred method of treating an ailment).
Internal workload	Individual physiological, psychological, or biomechanical response to an external load.
Load management	The process of quantifying and monitoring the exertional output of the athlete to mitigate overuse injuries and to improve overall health over a long-term period.
Negative predictive value	Percentage of athletes with a negative test who do not have an ailment Calculated as: (True Negative) /(False Negative + True Negative).
Physiological sensor	Sensor which measures metrics deemed critical to the internal workload of the athlete (e.g., heart rate, heart rate variability, muscle oxygen saturation).
Positive predictive value	Probability that athletes with a positive screening test truly have a condition (e.g., efficacy of a wearable device and its analytical platform to accurately diagnose an arrhythmia), Calculated as: (True positive)/(True Positive + False Positive).
Predictive analytics	Algorithms used to forecast the likelihood of a situation based on baseline data sets.
Return to participation	Process of deciding when an injured or ill athlete may safely return to training.
Return to play	Process of deciding when an injured or ill athlete may safely return to competition when the risk for re-injury is minimized.
Return to sport	Process of deciding when an injured or ill athlete may safely return to practice when the risk for re-injury is minimized.
Session rate of perceived exertion	Scale from 1 to 10 wherein the athlete subjectively rates the difficulty of each session. The product of the session difficulty and the session duration (in minutes) provides the “internal load” for that session.
Sensitivity	Ability of a test or model to correctly classify an individual as “diseased.” Calculated as: (True positive) /(True positive + False Negative).
Specificity	Ability of a test to correctly classify an individual as “disease- free.” Calculated as: (True Negative) /(True Negative + False Positive).
Sports scientist	Individual responsible for developing predictive analytical models and translating data from wearable sensors to derive value for the athlete, team physician, and athletic trainer.
Validity	Accuracy of a model; measure of the sensitivity and specificity of the model.
Wearable sensor	Device unencumbered by wires capable of detecting and translating biomechanical, physiological, and biochemical parameters to assess human health in a real-time manner.

## Five Parameters Measured by Wearable Sensors to Minimize Injury Risk in Athletes

The field of sports medicine has seen a rapid increase in the use of wearables over the last decade, much of it precipitated via advancements in data analytics, sensor fabrication technologies, and sports science (Seshadri et al., 2017, 2019a,b,c). Wearable sensors come in various forms ranging from wrist-monitors, epidermal patches, GPS and RFID sensors, chest/leg straps, and smart clothing, all of which measure many biomechanical or physiological variables, such as heart rhythm, heart rate (HR), heart rate variability (HRV), muscle oxygen saturation (SmO_2_), respiration rate (RR), and tri-axial acceleration (Bourdon et al., 2017) (Table 2). This review discusses the applications of wearable technology and analytics in optimizing athlete performance by monitoring athlete subjective exertional fatigue, movement profiles, hydration status, sleep, cardiac health, and monitoring SmO_2_ in a return-to-play (RTP) format following a traumatic injury (Table 3).

**Table 2:** Comparative analysis of wearable sensor modalities utilized in sports today.

**Technology**	**Capabilities**	**Pros**	**Cons**	**Leagues**
Surface EMG	Identifies muscle recruitment, potential weaknesses	Small processing unit, wireless; live data	Low Signal to Noise Ratio	MLB, NBA, MLS
GPS, RFID tracking, and Accelerometers	Distance, velocity, acceleration, deceleration, side-side movement, work output, sleep (actigraphy)	Easy to incorporate into football pads; vast amount of data points	No biometric data (HR, RR, EMG); price point; Limited per position (linemen or goaltenders). Must wear sport top as well as pod	NFL, NBA, MLS, European Football, NCAA Football, NCAA Basketball
ECG/PPG sensors	Heart rate, HRV, Respiration rate, stress levels, TRIMP, Muscle oxygen saturation	Accurate, cost effective, ease of use	Price point and accuracy of certain devices in question	NFL, NCAA, European Football, NBA, US Soccer, NFL

**Table 3:** Applications in sports medicine where the translation of wearable sensor data can be used to train predictive models toward monitoring the health and safety of athletes.

**Application**	**Clinical ailment**	**Data from sensors**	**Wearable SENSORS utilized in sports performance**
Workload management	– Over-use resulting in soft-tissue injury	– Distance – Velocity – Tri-axial acceleration – Muscle Oxygen Saturation	– Catapult OptimEye S5 (Li et al., 2020) – Zebra RFID (Zebra and The NFL, 2019) – Moxy Monitor (Crum et al., 2017)
Dehydration and soft-tissue injury prevention	– Soft-tissue injuries – Hypernatremia – Muscle strain – Fatigue	– Analyte Concentration – Sweat Rate – Whole-Body Sweat Loss	As of July 2020, there lacks current wearable sensor technology to monitor biochemical markers.
Sleep monitoring	– Fatigue – Athletic performance – Decreased reaction time – Executive functioning – Learning	– Heart Rate – Sleep quality – Tri-axial acceleration – Body Temperature	– WHOOP Band (Sekiguchi et al., 2019) – FitBit Flex; Charge2
Cardiac health	– Atrial fibrillation – Hypertrophic Cardiomyopathy	– Heart Rate – ECG signal – Blood Volume	– Apple Watch 4/5 (Seshadri Dhruv et al., 2020) – Wavelet Health Wristband (Dur et al., 2018)
Return-to-play	– ACL Tears – COVID-19	– Distance – Velocity – Tri-axial acceleration – SmO_2_ – Respiration Rate – Resting heart rate	– Catapult OptimEye S5 (Li et al., 2020) – Zebra RFID Chip (Zebra and The NFL, 2019) – Moxy Monitor (Crum et al., 2017) – WHOOP Band (Sekiguchi et al., 2019)

*SOC, Standard of Care; AF, Atrial Fibrillation; HCM, Hypertrophic Cardiomyopathy*.

### Example 1—Monitoring Subjective Rating of Perceived Exertion and Movement Profiles

Injury models began as linear paradigms, implying that events follow each other sequentially from a beginning to an end point (Meeuwisse, 1994). The nature of injury in sport is more complex, as injury exposure may occur with different intrinsic and extrinsic risk factors (Bahr and Krosshaug, 2005) or as a result of multiple participations without permanent removal from participation (Meeuwisse et al., 2007). If no injury occurs to the susceptible athlete, adaptation or maladaptation occurs through repeat participation. If injury occurs, it is either followed by recovery or removal from participation (Windt and Gabbett, 2017) (Supplementary Figure 1). Stern et al. paralleled athletes and their resistance to injury to a dynamic system like hurricanes; injury prediction methodology must more frequently and broadly sample parameters that factor into their injury determinants (Stern et al., 2020). Despite acknowledging many variables are at play, the center of most injury prediction models has been the assessment of an athlete's perceived exertional output and movement parameters such a distance, velocity, and acceleration.

**Figure 2:**
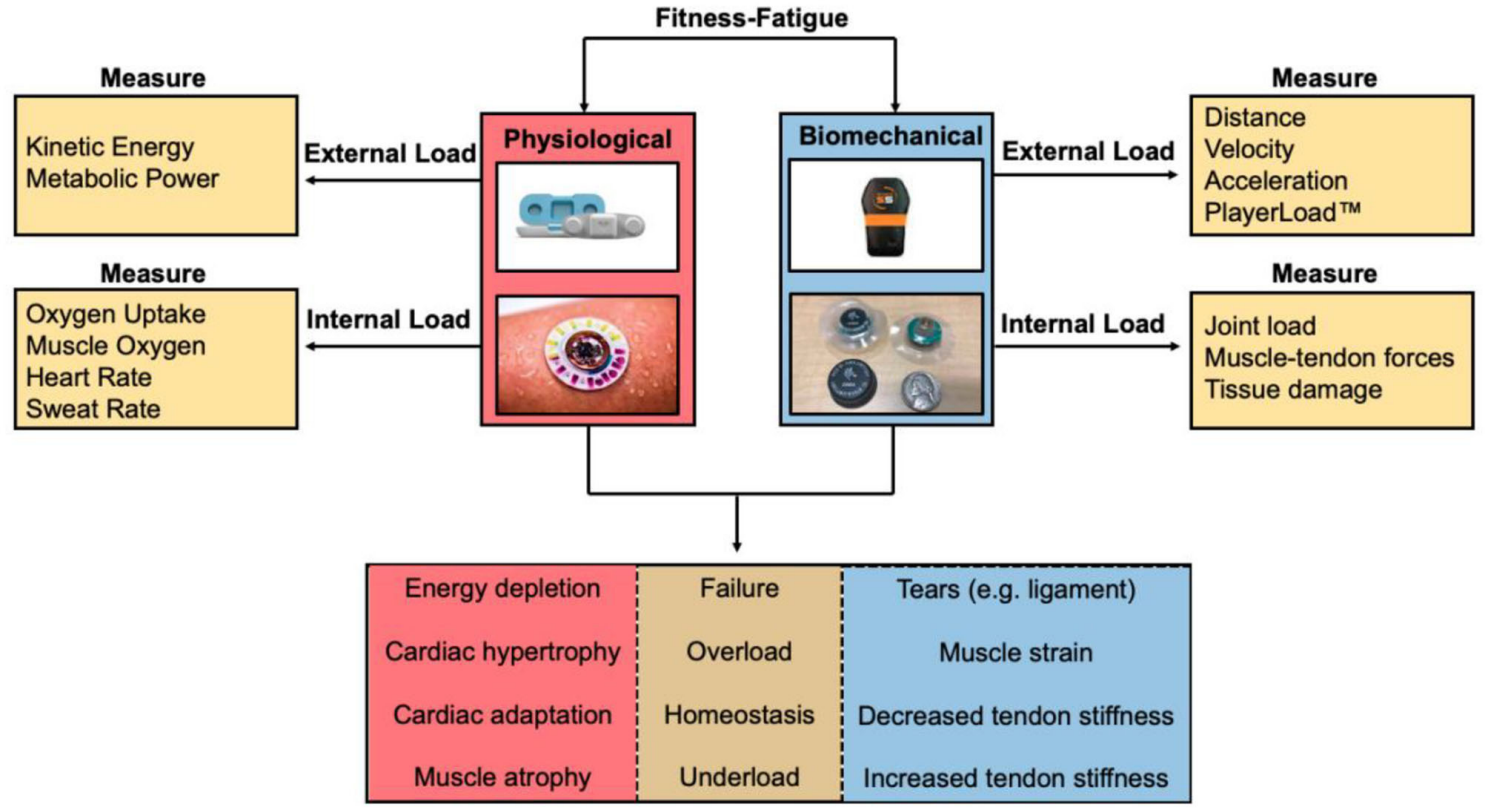
Fitness-fatigue relationship based on measuring physiological and biomechanical parameters from wearable sensors. Internal and external workload of the athlete can be determined to quantify the exertional output of the athlete to assess whether the athlete is training at an optimal level, undertraining, overtraining, or is at a considerable risk for injury.

**Figure 3:**
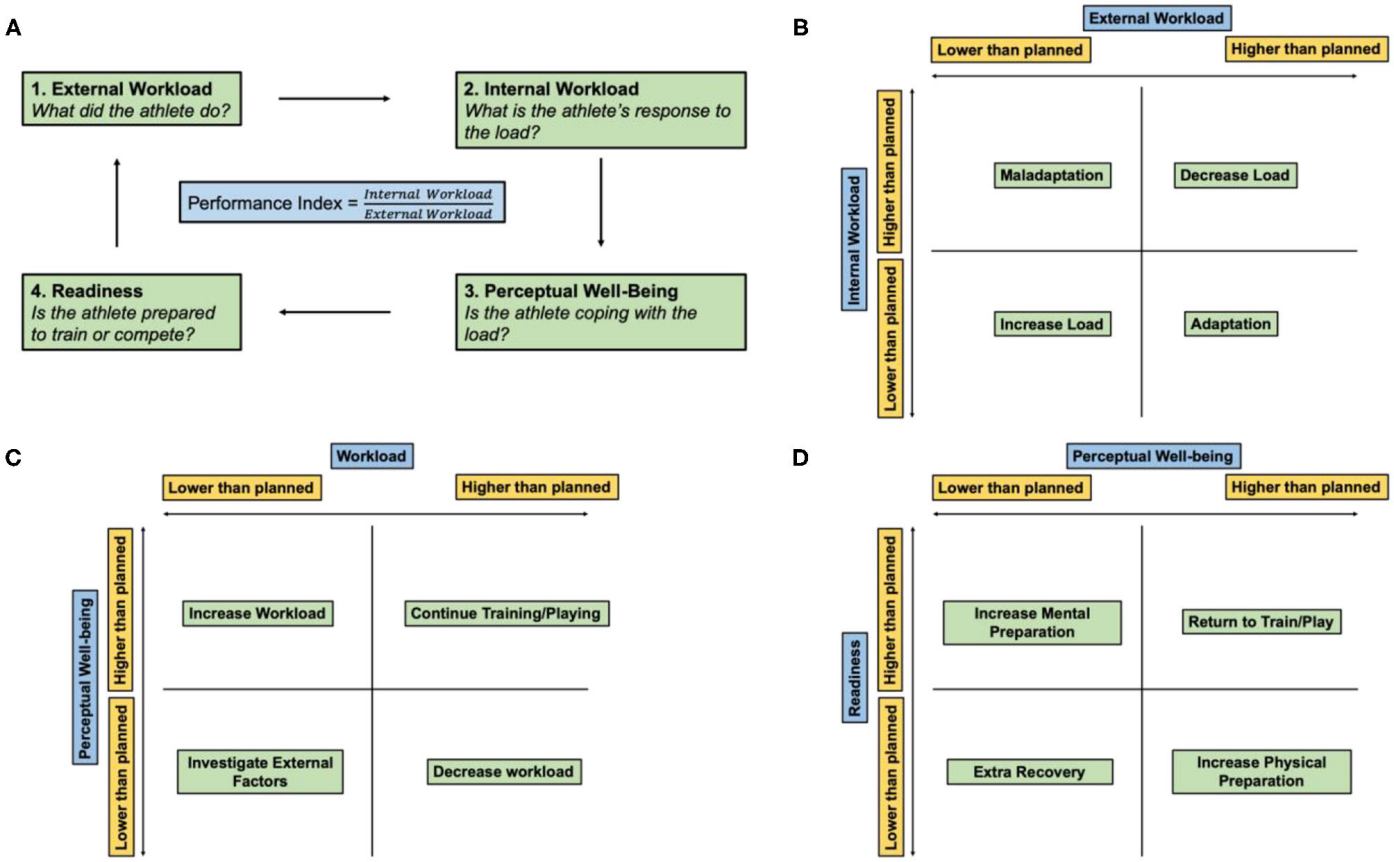
Athlete monitoring cycle. **(A)** Athlete performance index is the ratio of the internal and external workload of the athlete and encompasses how the athlete prepared, coped, and expended during a workout or match. **(B)** Relationship between the internal and external workload. **(C)** Relationship between the perceptual well-being of the athlete and overall workload. **(D)** Relationship between athlete readiness and perceptual well-being. Adapted and modified from Gabbett et al. (2017).

Parameters measured by wearable sensors can be used to calculate an athlete's external or internal workload during a training session (Seshadri et al., 2019c) (Figure 2). External workload is an objective measure of the demand on the athlete's body through measurements of movement measured by accelerometery or GPS (Seshadri et al., 2019c). Internal workload is defined as any physiological, psychological, or biochemical change in the body in response to external workload and includes metrics such as HR, muscle oxygen saturation (SmO_2_), and subjective rating of perceived exertion (sRPE) (Bourdon et al., 2017; Seshadri et al., 2019c). Developing effective methods to characterize workload profiles can inform an athlete's acute-to-chronic workload ratio (ACWR), an indication of how hard an athlete is training and their readiness to take on higher athletic demands (Blanch and Gabbett, 2016; Bowen et al., 2017; Murray et al., 2017). Furthermore, integrating internal and external workloads may provide a quantitative measure of an athlete's state of fatigue (e.g., sum of their internal, task, and environmental constraints) (Figure 3). Much of the current literature employing the ACWR has simplified the internal and external workload of the athlete to a function of sRPE and movement profiles, respectively.

“Load management” is the balancing of an athlete's recovery with their external (e.g., movement profile) and internal loading (e.g., perceived exertion, cardiovascular adaptation) to prevent abnormal training responses, such as overuse injuries. Overuse injuries occur due to repetitive submaximal loading of the musculoskeletal system due to insufficient rest, thereby preventing structural adaptation to take place (DiFiori et al., 2014; Jayanthi et al., 2015, 2020; LaPrade et al., 2016). Previous work has demonstrated an exponential relationship (~*r*^2^ = 0.53) between the ACWR using sRPE and movement parameters to assess workload and the probability of suffering an over-use injury (Hulin et al., 2014, 2016). This relationship was first explored in a study of cricket fast bowlers (Hulin et al., 2014). Load was monitored by calculating the sRPE, a psychophysical measure as a surrogate for the body's internal load, and counting balls bowled, an external measure that quantified motion and mechanical stress (Hulin et al., 2014). When the ACWR of the bowlers was <0.99, the resulting chance of injury over the next week was 4%. Alternatively, when the ACWR of the bowlers was >1.5, the risk of injury over the next week was 2–4 times greater. The authors concluded that loading spikes above an athlete's standard workload increased the risk of injury. The results demonstrated the utility of the ACWR to monitor athlete workload utilizing a simplified model of internal and external workload.

Today, the ACWR is a popular and commonly researched metric for injury risk stratification. A systematic review by Andrade et al. used 20 studies, totaling 2,375 injuries from 1,234 athletes in various sports, to investigate an association between ACWR and the risk of injuries resulting in loss of playing time in elite team sport athletes (Andrade et al., 2020). While the methodological approaches to calculating ACWR in the examined studies were heterogenous, the study justified the adoption of the ACWR, as the majority of studies suggested that athletes were at a greater risk of time-loss injuries with an elevated ACWR compared to a moderate or low ACWR using sRPE as a measure of internal workload and movement-parameters (or repetitive sport-specific activities in the case of the bowlers) as a measure of external workload. Of note, the review used a study sample of elite athletes that was composed entirely of male athletes (Andrade et al., 2020). Female athletes have an increased likelihood of select soft-tissue injuries (e.g., ACL tears) manifesting from repeated overuse (Webster and Hewett, 2018) so there remains a need to adapt current ACWR analytics to youth, collegiate, and elite female athletes (Arazi et al., 2020).

**Figure 4:**
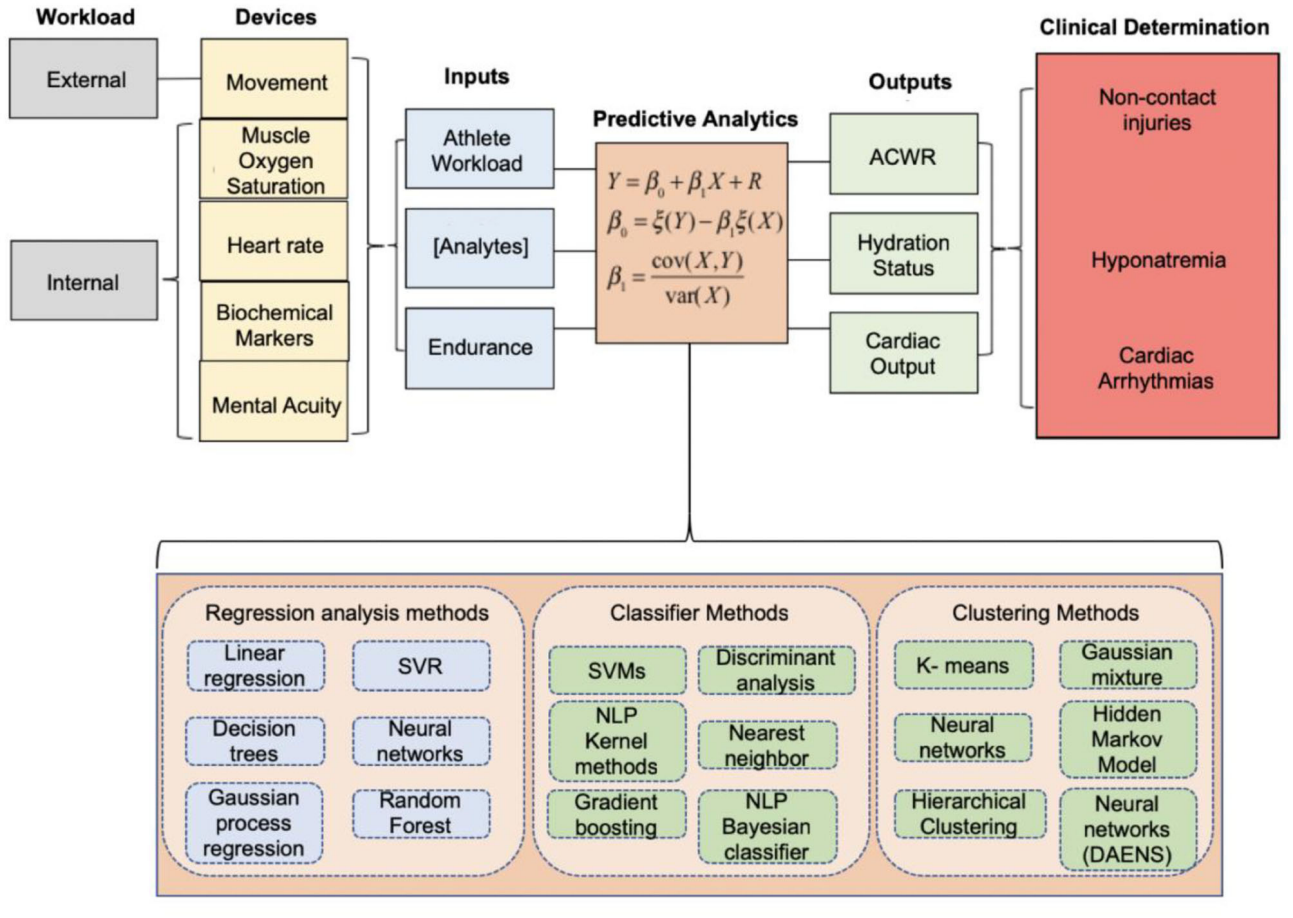
Integrated model proposed by our team at Case Western Reserve University and University Hospitals Cleveland Medical Center in 2019 to quantify the athlete's performance and health index. The data acquired from wearable sensors can be inputted into machine learning models to optimize athlete performance, assess the likelihood of suffering a non-contact injury, inform hydration status to alleviate soft-tissue injuries, or assess the cardio-respiratory function and capacity of an athlete. Adapted and modified from Seshadri et al. (2019c).

While stratifying injury risk based on a simplified model of internal and external workload using the ACWR model is deemed the “gold-standard” in sports performance today and has been validated by the International Olympic Committee (IOC) (Schwellnus et al., 2016; Soligard et al., 2016), it has not been universally accepted by sport scientists. Impellizzeri et al. created a statistical argument to call for the dismissal of the ACWR as a framework for “predicting injury” (Impellizzeri et al., 2020). Using sRPE data from a professional Italian Serie A football team, Impellizzeri et al. highlighted that the lack of predictive value of the ACWR stemmed from its tendency to conflate statistical significance and odds ratios. Performing such analysis with objective rather than subjective data is necessary to remove potential data bias provided by the athlete and confound results disseminating from the algorithm. Bornn et al. developed a simulation using daily training loads from Serie A (Italian Soccer) and National Football League (NFL) players. The authors simulated 1,000 player-seasons where the probability of injury depended only on player workload in the session of injury. Although the model was designed so the ACWR would not have an influence on injury risk, the simulation found a statistically significant relationship between injury risk and ACWR (where ratios were flagged if they fell outside of 0.8–1.3). These results imply the relationship between ACWR and injury is a confounded one; an athlete's training schedule may result in the significance found by studies correlating ACWR with injury risk (Bornn et al., 2019). Whether or not there is merit to the use of ACWR, there remains a need to improve and develop new multivariate models to more accurately establish a relationship between athlete workloads and overuse injuries (Bornn et al., 2019; Impellizzeri et al., 2020). While many studies regarding workload measure ACWR, the authors of this review recommend against the use of any one variable to stratify injury risk, including the ACWR. Application of a sport- and context-specific model is necessary and lacking to ensure a high positive predictive value as it relates to monitoring the health, performance, and wellness of athletes. An illustration of this theoretical model demonstrating the integration of all relevant physiologic metrics in determining athlete workload is shown (Figure 4). That being said, some studies have found associations of movement profile parameters with injury risk and have applied ML models to aid in injury prediction based on these parameters. Selected studies are discussed below focusing on youth, collegiate, and elite-level athletes.

Injuries related to overuse are of particular concern for a significant portion of the 3.5 million youth athletes (<18 years of age) injured in the U.S. annually (Ryan et al., 2019). Youth athletes remain the most under-studied population cohort relative to those within professional and intercollegiate sports (Jayanthi et al., 2013, 2015; DiFiori et al., 2014). There is clinical utility to assess the risk of soft-tissue injuries in youth athletes from repeated overuse by leveraging ML models to monitor the internal and external workload from wearable sensor data. Bowen et al. explored the association between workload and injury risk in youth elite male soccer players (age 17.3 +/- 0.9) who performed all training sessions and matches for two seasons with GPS/accelerometer units (Viper V.2, StatSports, Ireland). The sensor measured total distance, high-speed distance, and accelerations, and a binary logistic regression model was employed to compare workloads between injured and non-injured players (Bowen et al., 2017). Non-contact injury risk was greatest (RR = 5.11) when a very high number of accelerations were accumulated in 3 weeks (>9,254). Additionally, when greater high-speed distances (3,502–5,123 m above 20 km/h) were experienced in the setting of low chronic high-speed distance training over 4 weeks, a significant increase in injury risk was observed (RR = 2.55). These findings suggest that elite youth athletes may see a reduction in non-contact injuries by monitoring external workloads using GPS movement profiles to limit consistent and persistent acceleration efforts and by prescribing high-speed running distances in varying amounts over a set time period (e.g., 4 weeks). The application of ML to ascertain associations between workload and other variables with injury risk in youth athletes motivates research to address the “pandemic” involving early sports specialization.

At the collegiate level, Sampson et al. used GPS data from 52 NCAA football athletes and found that relative non-contact injury risks were 3x greater with an elevated ACWR compared to moderate and low ACWR (Sampson et al., 2018). Injury risk was similar across all positions. Substantially increased injury risk with a low chronic workload emphasizes the need for proper load management, especially in athletes with multiple game or training absences. Wellman et al. utilized the Catapult SPI HBU GPS sensor to monitor and compare movement profiles (distance, velocity, and acceleration) among various position groups in Division I American Football players to study the physical demands during competitive play (Wellman et al., 2016). Data showed that wide receivers and defensive backs had more high-intensity acceleration bursts and traveled greater distances compared to other positions. The findings suggested that predictive analytics using a one blanket detection method could vary in effectiveness from position to position, even within the same sport. Additionally, load experienced by linemen, a position which also involves heavy exertion with less running and high-intensity acceleration, may not be accurately represented by GPS. GPS data from linemen, and any athletic position where players experience high stress loads constrained to a small space, will be limited to recording positional data without accounting for the mechanical load experienced by musculoskeletal tissue (Edwards et al., 2018). These results highlight the need for sport, and even position-specific metrics that optimize injury risk stratification models (Gastin et al., 2014; Chambers et al., 2015; Wundersitz et al., 2015). Like linemen, an accurate workload assessment of the athlete would require a combined use of both internal and external workload. Such work in the sports medicine field has yet to be published and could greatly enhance the translational utility of such devices.

On the professional level, members of this team studied the effects of player workload measured by the Catapult OptimEye S5 and its proprietary algorithm using tri-axial acceleration on soft tissue injuries in a single NFL team over two seasons (Li et al., 2020). Defining acute and chronic workloads as 1 and 4 weeks prior to injury, the authors found that an athlete with an ACWR above 1.6 was 1.5 times more likely to sustain a myotendinous or ligamentous injury. Injured players saw a 111% increase in workload during the week of injury while uninjured players were found to have a 73% increase in workload. Like the collegiate study, this study further emphasizes the workload-injury risk association using athlete movement profiles. To take a step further, multiple studies have investigated ML techniques to detect professional athletes at high-risk of injury using workload as the main injury determinant. These prediction models, motivated by the data suggesting a workload-injury risk association, apply data processing techniques and ML to estimate injury probability (Seow et al., 2020). Colby et al., investigated lower body non-contact injuries in professional Australian football athletes and measured sRPE and GPS-derived total distance, sprinting distance, and maximal velocity over three seasons and found that minimal athlete exposures to high velocity efforts (85% maximal velocity) over the previous 8 weeks were associated with significantly greater injury risk than athletes with a greater number of high velocity efforts (Colby et al., 2014). Injury risk increased additionally for athletes with many repeated exposures to high velocity sessions. The study suggested exposure to >85% of an athlete's maximum velocity over 5–8 sessions (training or competition) over a 4 –weeks block minimized the predicted probability for injury, with the greatest predictive accuracy using a Poisson log-link regression model (AUC range = 0.60–0.64) (Colby et al., 2014). In another study, Rossi et al. attempted to build an effective injury forecaster for an elite-level Italian soccer club, using 10 Hz GPS devices integrated with a 100 Hz 3D accelerometer, a 3D gyroscope, and a 3D digital compass (STATSports Viper) paired with ML platforms (Rossi et al., 2018). The forecaster employed a Recursive Feature Elimination with Cross-Validation (RFECV) to determine which subset of features was most effective at predicting injury, and a decision tree binary classifier method to predict either injury (1) or no injury (0) for a given athlete's incumbent training session. The model was built with a set of training data containing 55 different features from 952 different examples (e.g., training sessions with a known output as “injured” or “not-injured”). The predictive model accounted for various features, including data from the GPS sensors, metabolic data (e.g., metabolic distance, high metabolic load distance, and high metabolic load distance per minute), previous workload history, ACWR, and mean to standard deviation workload ratio (monotony). The study concluded that the decision tree-model was more effective – with 80% sensitivity and 50% specificity in cases of injury – than state-of-the-art controls, such as ACWR modeling – with 91% sensitivity and 4% specificity – at identifying athletes who were at risk for injury (AUC = 0.76) (Rossi et al., 2018). Lastly, Carey et al. investigated the occurrence of hamstring injuries in professional Australian football athletes using GPS movement data over two seasons (Carey et al., 2018). Injury predictions were generated during the third season and employed various prediction models (regularized logistic regression, generalized estimating equations, random forests, and support vector machines) to determine which technique had the best performance. Interestingly, the best performing model was the multivariate logistic regression model (AUC = 0.76), and models for total non-contact injuries with resultant time loss from competitive play had a predictive performance only marginally better than chance (AUC < 0.65). One may conclude that models of the relationships between GPS workload and non-contact injuries showed limited ability to predict future injuries; however, given the low injury rate per session (0.4% for hamstring to 3% for all non-contact) and the large number of possible risk factors, a probabilistic approach to injury risk models using workload should be employed and may result in better prediction power in future studies. The results of this study suggest that specific injury types as outcome variables may reveal better prediction power that general non-contact models and that increasing the quantity of data will likely yield better predictive performance. Importantly, movement-profile driven workload calculations as a daily decision tool has limited ability to predict injury; over longer periods of time, the predictive performance of using a probabilistic model (logistic or Poisson regression) may provide a better assessment of injury likelihood. At present, the performance of injury prediction models with sRPE and movement profiles as the primary injury determinants are poor, with a median AUC of the best performing models being 0.75 (Hulin et al., 2016). This is likely attributable to small samples sizes, and, more importantly, an incomplete understanding of the relevant injury-determinant factors used in the models for workload. However, knowing there are associations with movement profile variables (ACWR, consistent acceleration efforts, etc.) and injury risk, future studies may seek to investigate larger and heterogeneous cohorts of athletes and perform prospective randomized studies.

### Example 2—Monitoring Athlete Hydration Status

Monitoring sweat rate and electrolyte levels has applications in monitoring the performance of athletes. Alterations in the electrolyte composition in sweat and sweat rate can act as indicators for hydration levels and provide insight into the athlete's physiologic state. It is well-established that sweat rate is proportional to exercise intensity (Baker, 2017). This relationship is also uniquely substantiated in youth athletes, where youth American football players have been shown to sweat at two times the rate during competitive games than during practice (Baker, 2017). Baker and colleagues used epidermal sweat patches to quantify sweat and electrolyte losses of athletes during training (Baker et al., 2009, 2016; Baker, 2017). Several studies have identified sweat and blood lactate to be a potential marker for physiologic stress during exercise, capable of tracking exercise intensity by marking transitions in physical stress from aerobic to anaerobic exercise (Graham et al., 1987; Jia et al., 2013). Once a certain lactate threshold is reached, exercise capacity is greatly reduced, and the athlete is subject to exhaustion (Graham et al., 1987; Katz and Sahlin, 1988). Sweat glucose levels have also been shown to model blood glucose levels, a prime factor in exertional capacity (Count et al., 2019). A drop in blood pH levels may indicate insufficient supply of glucose or impaired lactate clearance, therefore monitoring decreases in sweat pH can also assist in an assessment of the patient's metabolic state (Yokus et al., 2020). All in all, these studies suggest that an athlete's physiologic capacity and efficiency under physical stress can be measured with sweat analyte composition, which provides us with another tool to better understand an athlete's internal workload.

Training datasets need to be created from clinical trials that track a large array of physiologically relevant features (e.g., whole body sweat loss, changes in ion concentrations from eccrine sweat) to label cases when an athlete experiences dehydration (1) or did not experience dehydration in the training session (0). At present, the focus of the current literature is on the development and validation of novel wearable systems and producing a durable device that can provide real-time multi-parameter analysis of sweat during exercise. The authors anticipate that over the next several years, large prospective observational studies will be conducted using both novel and commercially-available devices on athletes such as the Gatorade Gx Sweat Patch, which can measure sweat rate and sodium chloride concentration. In these future studies, a form of dimensionality reduction, paired with a predictive classifier such as a LASSO technique could help assess the complex relationships between physiological determinants of hydration status. Deep neural networks, which focus on analyzing complex patterns in data structures, also offer promise as an automated hydration status analyst. If a model is able to predict future cases of dehydration with relative accuracy, further research efforts can be implemented to predict the incidence of soft-tissue injury from deviations in key eccrine sweat ion concentrations (Table 4).

**Table 4:** Machine learning models currently utilized in sports medicine toward monitoring the health and safety of the athlete.

**Model classification**	**Model description**	**Application in sports medicine**
Decision Tree Classifier (DTC)	Decision Tree models are used in both regression and classification.	Classify whether athletes will or will not sustain an injury in their training session (Rossi et al., 2018)
Dimensionality reduction	Dimensionality reduction is a technique used in supervised ML models that indexes the importance of feature subsets by calculating each's features correlation with the outcomes provided in training data. It can be used to eliminate indeterminate features.	Identify important features in a study analyzing 63 parameters, determining only three were needed to form a predictive model (Yokus et al., 2020)
Least Absolute Shrinkage and Selection Operator (LASSO)	The LASSO method provides identification of non-linear parametric models by selecting a subset of features that are most associated with the response variable. The algorithm accomplishes this by minimizing the residual sum of squares leading to the determination of correlation coefficients. Variables with the highest correlation coefficients to specific outcomes will be used to estimate the structure of a non-linear polynomial predictive model (Bumgarner et al., 2018)	*Assess relationships between physiological determinants of hydration status by stratifying changes in eccrine sweat ion concentrations with changes in external workload profiles
Logistic regression	Model the probability of a certain class or event existing such as pass/fail, win/lose, alive/dead, or healthy/sick	Assess the relationship between SRPE/movement profiles and soft-tissue injury (Carey et al., 2018)
Multi-layer perceptron (“neural network”)	A Multi-Layer Perception can be either a classification or regression model. Features are transformed throughout a series of hidden layers. The first layer are the inputs, or features given in the dataset, in our case, from the athlete. An MLP with one hidden layer can estimate any continuous bound function, while an MLP with two hidden layers can estimate any function, within a small error provided enough quality training data.	*Assess relationships between physiological determinants of hydration status by stratifying changes in eccrine sweat ion concentrations with changes in external workload profiles
Support Vector Machine (SVM)	A support vector machine is a binary classifier that graphs labeled data points in a two-dimensional plane. The algorithm then looks to separate the two labels with an optimized hyperplane. When new, unlabeled data is presented, each point is classified by which side of the classifying line it lies on.	*Forecast the workload profiles of an athlete based on position group, body mass index (BMI), or previous workload data to help mitigate soft-tissue injury or illness

The authors have provided applications or have hypothesized (the latter denoted by an ^*^) the application of the described model for sports medicine as it relates to monitoring the performance and safety of the athlete.

### Example 3—Monitoring Sleep in Athletes

The effects of sleep quality on athletic performance and injury rates is a burgeoning area at the intersection of psychiatric and sports medicine research. Athletes tend to sleep less and have poorer quality of sleep than non-athletes (Fietze et al., 2009; Sargent et al., 2014), which may be due in part to their unique scheduling constraints from training schedules, travel to competitions, and pre-competition anxiety (Sargent et al., 2014; Juliff et al., 2015). The effects of sleep deprivation include a multitude of physical and mental manifestations resulting in poor athletic performance (Simpson et al., 2017). In elite youth athletes, those who consistently slept more than 8 h during weekdays reduced their odds of injury by 61% (OR: 0.39; 95% CI, 0.16–0.99) (von Rosen et al., 2017). Additionally, prior research on NCAA Division I and III cross-country runners found the strongest predictor of athletes sustaining a new injury was poor sleep quality, alongside the existence of a pre-season injury or large mileage increase (Hayes et al., 2019). These findings demonstrate enormous clinical utility and performance-based incentive for the sports physician and athletic trainer to optimize sleep in the athlete.

Sleep-wake cycles are largely self-reported (Fox et al., 2020). Wearable devices that provide objective data toward accurately measuring sleep duration and quality are needed (Colbert et al., 2011; Peake et al., 2018). Wearable actigraphy devices are wristbands that monitor a combination of variables such as movement, HR, and HRV as indicators of sleep-wake cycles. While actigraphy devices are the most widely used sleep sensors (Van de Water et al., 2011), their reliance on motion to assess sleep tends to overestimate sleep and underestimate wakefulness across the sleep period (i.e., high sensitivity and low specificity for sensing sleep) (de Zambotti et al., 2015). A review by Scott et al. compared the accuracy of wearable actigraphy devices to polysomnography and found that the Pearson correlations ranged from *r* = 0.01 to *r* = 0.73 (Scott et al., 2020). A criticism of any device that detects behaviors of interest is they generally become validated in laboratory settings with a limited number of activities of interest (Intille et al., 2012; Welch et al., 2013). This led to work by Ellis et al., where a multi-level classifier using random forest and Hidden Markov model was employed to identify activities performed by the wearer with no prior knowledge about their habits. They were successful in generating an activity classifier that reached 85.6% accuracy (Ellis et al., 2014). Inclusion of ML algorithms with sensor hardware are therefore an encouraging next step in the development of more accurate actigraphy sensors. While published literature has predominately investigated the efficacy of wearable devices for sleep detection, there remains a need to track changes in athletic performance and its implications on injury risk following an athlete's use of wearable sleep sensors. Randomized controlled trials using wearable sensors should be employed to bridge this gap to objectively monitor an athlete's sleep duration and quality to provide sports practitioners another variable toward reducing injury burden.

### Example 4—Monitoring Cardiac Health in Athletes

It is critical to distinguish between physiological and pathological adaptation of the heart to exercise (Baggish et al., 2017). A number of physiological, morphological, and functional adaptations occur in the heart secondary to regular, strenuous exercise which may be difficult to distinguish from pathological processes. While exercise is beneficial for most cardiac conditions, athletes with underlying heart conditions paradoxically carry an increased risk of sudden cardiac death (SCD) during strenuous exercise when compared to their sedentary counterparts (touchCARDIO, 2020). Pre-participation screening strategies to identify sub-clinical cardiac conditions (Maron Barry et al., 2014) remains highly controversial, with no agreed upon strategy. The combined use of ML with wearable technology has the potential to help screen and monitor cardiac physiology and pathology in the recreational and elite athlete as it applies to both performance and athlete safety.

Hypertrophic cardiomyopathy (HCM), an inheritable condition characterized by myocardial hypertrophy without systemic etiology, increases the risk of heart failure, stroke, and SCD (Captur et al., 2020). HCM is reported in pathologic registries as one of the most common causes of SCD in youth athletes (Rasmusen and Schmied, 2020), and HCM has been shown to be the most common cardiomyopathy in elite soccer players (Malhotra et al., 2018). Estimates of the prevalence of HCM in the United States predict only 16% of cases are clinically diagnosed (Maron et al., 2016), demonstrating the need for improved detection of HCM. This could potentially be provided with ML and wearable sensor technology using predictive analytics in undiagnosed athletes of all ages and ability. Green et al. theorized that patients with obstructive HCM (oHCM) could be distinguished from controls using a combination of machine learning and the photoplethysmography (PPG) capabilities of a commercial biosensor (the Wavelet Health wristband) (Green et al., 2019). By detecting blood volume changes at the skin surface, the optical sensor continuously recorded pulse wave traces of 19 oHCM patients (57.5 years ± 14.3, 47% female, interventricular septal thickness 1.64 ± 0.2 cm) and 64 healthy controls (28 years ± 7.4, 38% female, interventricular septal thickness 0.83 ± 0.13 cm). A set of 38 pulse wave morphometric features were found to significantly differ between groups which were used to develop an automated machine learning classifier for distinguishing between them. The model achieved a C-statistic for oHCM detection of 0.99 (95% CI: 0.99–1.0), a sensitivity of 95%, and a specificity of 98%. The model was able to correctly classify 18/19 oHCM patients and 63/64 healthy volunteers with 98% accuracy (Green et al., 2019). These results imply wearable biosensors accompanied by predictive analytics could assist in accurately monitoring cardiac physiology and potentially identifying cardiac structural pathology in athletes.

The integration of ML algorithms into FDA-approved wearable ECG devices (including the Apple Watch 4, AliveCor KardiaBand, and AliveCor Kardia Mobile) has emerged as a non-invasive method to detect and monitor cardiac rhythm (Table 5). Monitoring of cardiac arrhythmias such as atrial fibrillation (AF) has additionally been shown to be crucial in monitoring the cardiac health of both retired and current athletes (Turagam et al., 2015; Estes and Madias, 2017). A recent study showed that retired NFL athletes (age 56 ± 12 years, 47% African American, *n* = 460) had a 5.5 times higher likelihood of developing AF than individuals from the general public with similar demographics (age 53.9 ± 8.6 years, 53% African Americans, *n* = 925) (Aagaard et al., 2019). Most of the former players were unaware of their AF diagnosis prior to the study, thereby demonstrating the importance of long-term medical follow-up to abnormal heart rhythm in such higher risk populations (Aagaard et al., 2019).

**Table 5:** Digital health devices (e.g., wearable and hand-held) currently studied by sports cardiologists to monitor the cardiovascular health and wellness of athletes.

**Feature**	**AW 4/5**	**Qardiocore**	**Alivecor KardiaMobile (1L/6L) or KardiaBand**	**Hexoskin**	**Zio patch**	**Holter monitor**
Wearable device	Yes	Yes	Yes (KardiaBand)	Yes	Yes	No
Auto alarm	Yes	No	Yes	No	No	No
1-lead ECG	Yes	Yes	Yes	Yes	Yes	Yes
6-lead ECG	No	No	Yes	No	No	Yes
Mobile application	Yes	No	Yes	Yes	No	No
Continuous monitoring	No	Yes	No	Yes	Yes	Yes
Real time analysis	Yes	No	Yes	No	No	No
Employs ML	Yes	No	Yes	No	No	No
Accuracy of ML (%)	95.8% (TP) (Turagam et al., 2015) 97.7% (TN) (Turagam et al., 2015)	–	96.6% (TP) (Aagaard et al., 2019) 94.1% (TP) (Aagaard et al., 2019)	–	–	–

*Information gathered from company websites, social media sites, and press releases. “N/A” implies that accuracy of device has yet to be validated in peer-reviewed studies against a 12-lead ECG to monitor atrial fibrillation*.

*1L/6L, 1-Lead or 6-lead; TP, True-Positive; TN, True-Negative; AW, Apple Watch*.

The Apple Heart Study (AHS) evaluated the accuracy of generated tachograms (derived from the Apple Watches 1–3) in detecting new AF cases (Turakhia et al., 2018). While results of the AHS have many limitations, the study successfully used ML to detect AF and provided initial evidence demonstrating the utility of wearable sensors to monitor AF. Apple Watch technology also allowed for the ability to successfully enroll and monitor patients virtually for a site-less study (Turakhia et al., 2018). Future studies should aim to investigate the relevance of the junction between ML algorithms and wearable ECG sensors from trials without the limitations of the AHS. Ultimately, these devices may allow for the continued monitoring of heart rhythm in athletes of all ages and ability.

In a study evaluating the accuracy of the AliveCor Kardia Band (KB), Bumgarner et al. examined whether the KB could differentiate sinus rhythm (SR) from AF compared to physician-interpreted 12-lead ECGs and KB recordings wherein the electrophysiologist was blinded to KB recordings(Bumgarner et al., 2018). One hundred consecutive patients (age: 68 ± 11 years) with AF presenting for cardioversion (CV) were enrolled. Patients underwent pre-CV ECG along with a KB recording. If CV was performed, a post-CV ECG was obtained along with a KB recording. Overall, compared to the ECG, the KB interpreted AF with 93% sensitivity, 84% specificity and a κ coefficient equal to 0.77. Physician-interpretation of KB recordings demonstrated 99% sensitivity, 83% specificity and a κ coefficient equal to 0.83. In short, the team concluded that the KB ML algorithm for AF detection, supported by physician review, can accurately differentiate AF from SR (Bumgarner et al., 2018). Future studies that utilize wearables to identify cardiac pathology should employ ML algorithms that can discriminate likely normal cardiac adaptations in athletes such as those described by Gray et al. (touchCARDIO, 2020), thereby reducing their false positive rate. Recent work in cardiac screening in Pacific-12 Conference institutions Division I collegiate athletes from 2009 to 2017 prior to the start of a season found that the use of an ECG compared to history and physical examination improved the cost-efficiency per diagnosis 5-fold (Harmon et al., 2020). The use of an accurate wearable ECG monitor may further drop the cost of diagnosis by enabling remote monitoring capabilities and expediting treatment.

### Example 5—Monitoring the Rehabilitation of Athletes Following ACL Reconstruction

**Figure 5:**
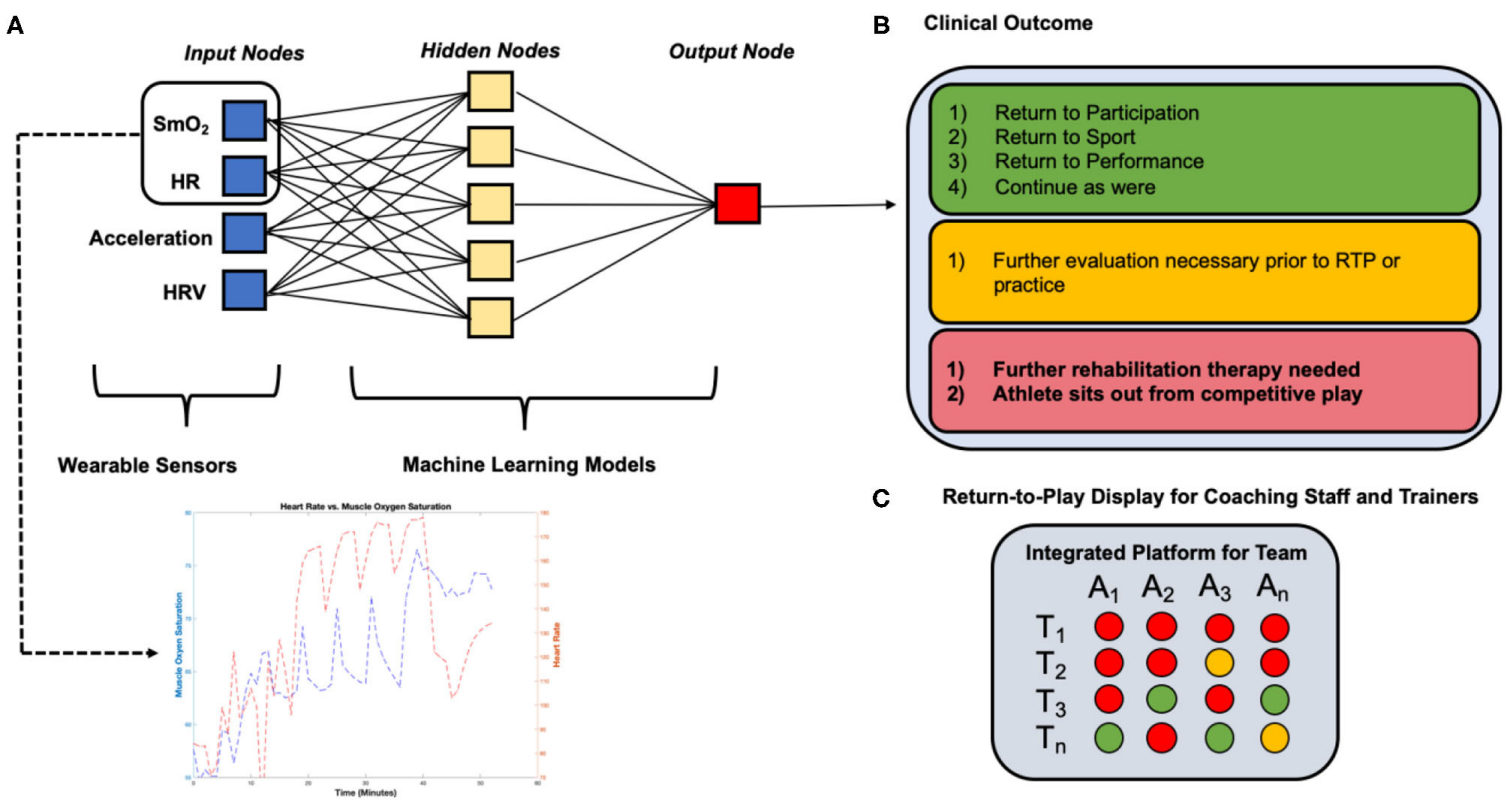
Example detailing the application of wearable sensor technology and machine learning algorithms employed in a Return-to-Play rehabilitation program following ACL reconstruction. **(A)** Wearable sensors collect continuous muscle oxygen saturation and heart rate data during a moderate-intensity quadriceps-focused exercise at a 12- weeks (T_1_) post-ACL reconstruction conditioning assessment during an athlete's RTP rehabilitation program, **(B)** machine learning algorithms process data collected from wearable sensors and determine a clinical outcome “forecast” based on learned population data, **(C)** coaching staff and trainers can track the athlete's readiness to return to competition over time and compare to other athletes, focusing the decision on the basis of analytics rather than intuition or subjective assessments.

Return-to-Play (RTP) protocols form the cornerstone of the rehabilitation and recovery period following a major musculoskeletal injury (Shrier, 2015; Shrier et al., 2015; Blanch and Gabbett, 2016; Musahl et al., 2018). Many common injuries such as an ACL tear (Musahl and Karlsson, 2019), hip arthroscopy (Ishøi et al., 2018; O'Connor et al., 2018), or Tommy John Surgery (TJS) (Jack et al., 2018) have a protocol that involves a period of rest followed by physical therapy until an athlete is able to resume sport-specific training and eventual competitive play. These injuries carry a known substantial burden on professional athletes (Dodson et al., 2016; Secrist et al., 2016), and ACL tears affect more than 200,000 people in the United States each year with direct and indirect costs >$7 billion annually (Musahl and Karlsson, 2019). Our discussion in this section will focus solely on providing insight into how wearable data and ML models can complement RTP protocols for an ACL tear following reconstructive surgery (Figure 5).

Rehabilitation from ACL reconstructive surgery currently includes a general consensus of 9 months before returning to sport, and extensive physical therapy beginning 1–4 weeks post-operation (Zaffagnini et al., 2015). Most sports medicine professionals use other physical assessments of the athlete to determine readiness to resume competitive play, yet re-injury rates remain high, suggesting that there remains a gap between the athletes' perceived vs. actual sports readiness. Dodson et al. evaluated the effectiveness of RTP practices following ACL tears from 2010 to 2013 in the NFL, which determined that 18.9% of players returning from injury experienced re-injury to the graft or contralateral leg (Dodson et al., 2016). These results suggest that rehabilitation practitioners could do a better job rehabilitating athletes, and there is an opportunity to complement current RTP protocols with more robust quantitative assessments of athlete readiness and better individualize the athlete's day of return to competition (Wiggins et al., 2016; Paterno et al., 2017; Losciale et al., 2018). One area to improve is highlighted by the association of re-injury with reduced quadriceps strength and power following ACL rehabilitation (Mueller et al., 2014). Despite aggressive rehabilitation programs directed at improving quadriceps function, a universally effective approach to reverse this weakness has not been fully elucidated. Furthermore, many ACL recovery programs use surrogate measures for quadriceps recovery including circumferential bulk measurements, closed and open kinetic chain testing, functional jump tests, and sport-specific testing; however, their validity in assessing appropriate time to return-to-sport with proven re-injury protection benefits is, at present, limited. We believe that wearable technology could play a role by being able to (1) collect data at a higher sampling frequency, (2) measure more clinically important markers of quadriceps recovery, and (3) estimate readiness to RTP.

Recent research has suggested RTP metrics must be expanded from performance (e.g., maximum running speed) prior to injury (Whiteley et al., 2020). Wearable devices measuring biochemical markers of muscle metabolism, oxygenation, and blood flow could therefore provide important insight into the rehabilitating quadriceps muscle. Specifically, a wearable near-infrared spectroscopy (NIRS) sensor such as the Moxy Monitor (Moxy Monitor, 2020) has the ability to measure SmO_2_ levels. This sensor can assess muscle oxygen utilization behavior in a continuous fashion during aerobic and anaerobic training conditions, providing a more comprehensive assessment of muscle recovery and function than any single clinical test. With SmO_2_ data collected during standardized exercise programs and new long-term clinical data on performance and re-injury after return-to-sport, ML algorithms can determine the SmO_2_ trend characteristics that are associated with optimal readiness to return-to-sport. Combining this metric with other metrics, including velocity, acceleration, HR, etc. during the rehabilitation program would add further sophistication to the model (Figure 5). However, we are beginning to understand that injury “prediction” requires more than a concentration on the risk factors associated with injury occurrence. Future models should seek to better understand how these variables influence each other in a dynamic system that is constantly changing to its environment. In an RTP setting; however, the stresses to the “athlete system” are prescribed and controlled by design, making it different from the competing athlete. The focus is to optimize functional parameters prior to re-engaging the athlete in competitive play. In this setting, investigating parameters (e.g., SmO_2_) that restore the athlete to their pre-injury performance level may prove to be effective in re-injury prevention. Little is understood about which functional parameters are the most important to monitor during rehabilitation and future clinical trials may seek to better understand these to prevent inappropriate clearance to return to competition.

## Current and Emerging Focal Point: Monitoring the Return-to-Play Status of Athletes Following COVID-19

**Figure 6:**
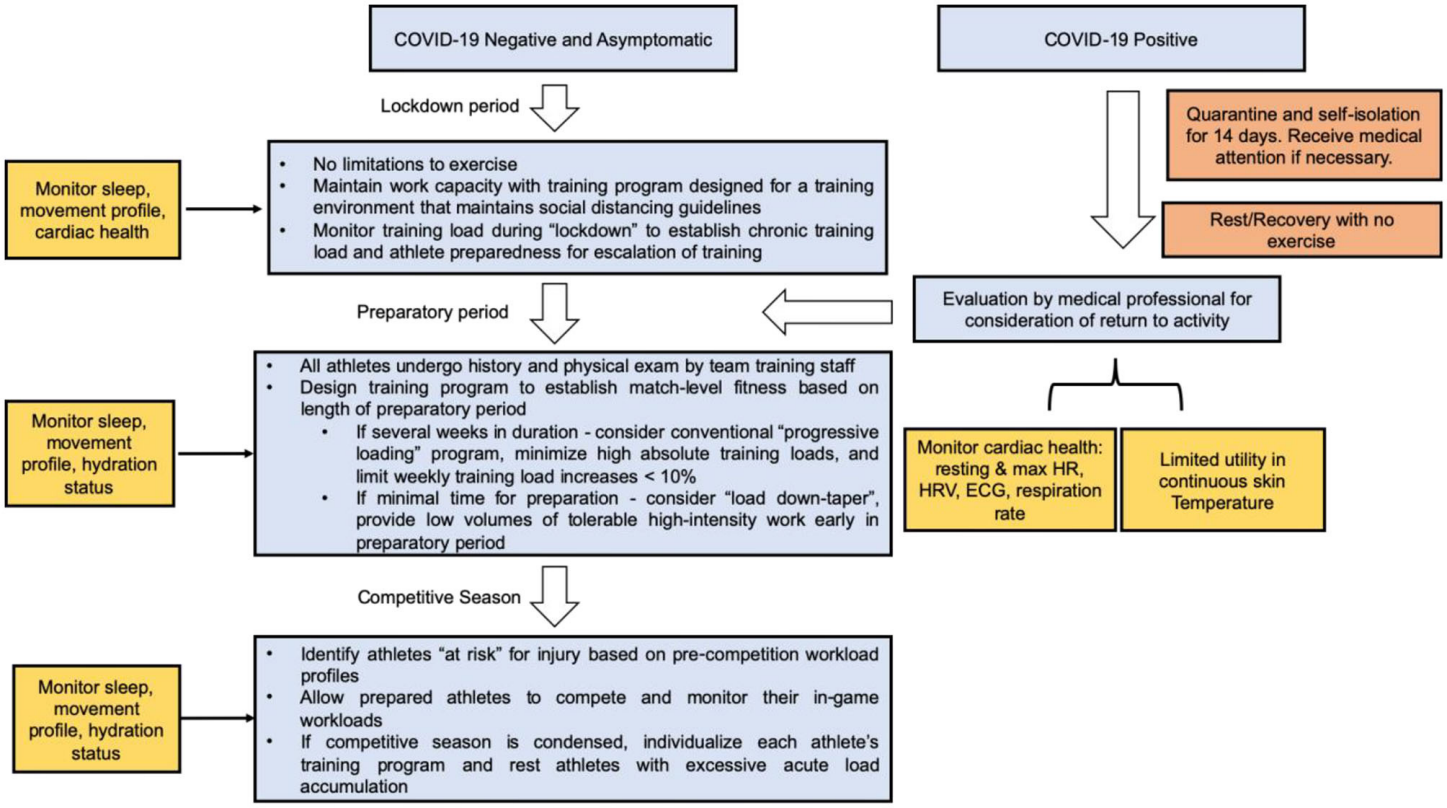
Role of wearable technology in a COVID-19 Return-to-Play algorithm for athletes of all ages. Continuous data collection using wearable technology can assist in maintaining and resuming training workloads following lockdown or athletes recovering from COVID-19.

As athletes begin to RTP following public health policy recommendations surrounding COVID-19, clinicians will be charged with determining when athletes are medically cleared, whether or not they have been infected. The lockdown period has been uniquely problematic for athletes due to the closure of training facilities worldwide. As athletes RTP following the COVID-19 lockdown, monitoring their internal and external workload is imperative toward assessing their biomechanical and cardiovascular adaptations with increased exercise intensities (Figure 6). The heterogeneity of training intensities and durations suggests that athletes will either undertrain, over-train, or train at an optimal workload relative to their training intensities during the pre-lockdown period. Based on trends in the literature (Gabbett, 2010; Blanch and Gabbett, 2016; Bowen et al., 2020; Myers et al., 2020), we hypothesize that the incidence of injury will be primarily bimodal, particularly evident in the athletes that over- and undertrain relative to pre-lockdown intensities. The undertraining cohort will suffer injuries (oblique, groin, and hamstring strains) manifesting from the lack of progression in chronic workload. The overtraining cohort will suffer injuries manifesting from muscular and neuromuscular fatigue, which has been known to increase the incidence of ACL tears in youth athletes (Fidai et al., 2018) or Achilles ruptures in professional NFL athletes (Myer et al., 2011). We hypothesize that the cohort who optimizes their overall fitness-fatigue relationship will reduce their risk of soft tissue injury as described in our prior models.

With the advent of the COVID-19 pandemic, it is important to acknowledge that ~22% of patients hospitalized with COVID-19 show signs of acute cardiac injury (ACI) in comparison to 1% of patients in non-COVID-19 viral infections. Wearable devices have shown to be useful in monitoring patients at risk for developing long-term sequelae of COVID-19 (Mishra et al., 2020; Seshadri et al., 2020). ACI can be detected using ECG readings, in conjunction with troponin levels and abnormalities on TTE (Driggin et al., 2020). An RTP algorithm recommended by Phelan et al. advises close cardiac monitoring of competitive athletes and highly active people (Phelan et al., 2020). Wearable devices, while only one piece of the diagnostic puzzle, may therefore be employed in the sports cardiologist's toolkit in the close monitoring of the returning athlete's workload measures and signs of cardiac pathology (Seshadri et al., 2020).

## Conclusions

The collection, agglomeration, and implementation of baseline datasets into analytic models based on data acquired from wearable sensors provide a vast toolkit for team physicians, athletic trainers, and sports scientists to make real-time decisions relevant to the health and wellness of athletes. Utilizing such baseline datasets coupled with analytical models now permits sports scientists, data scientists, and medical team personnel to work together to develop efficacious models to track the long-term health of athletes. The application of analytics in sports medicine when leveraging data from wearable technology will have—and is having—the greatest clinical impact when it comes to monitoring and forecasting the workload and cardiovascular health of the athlete, monitoring the sleep quality of the athlete, assessing the hydration status of the athlete to prevent hydration-related injuries, and complementing current RTP protocols. Future research involving the application of wearable sensors toward achieving high fidelity performance optimization models should focus on assessing the relationship between external and internal workload, thermoregulatory response, and biomarker concentrations (e.g., lactate, cortisol, creatine kinase) during dynamic exercise.

Can we assess the likelihood of injury using the intersection of wearable technology and ML? Currently, the answer still remains no. Current studies show that injury prediction is limited due to lack of accurate multivariate probabilistic models which are likely derived from an incomplete understanding of the determinants of injury and how they behave in a complex system. Rather than focusing on risk factors and associations, we are beginning to understand that athletes are dynamic systems that require a systems-based approach to understanding athlete states (in-competition, rehabilitating, etc.) and the network of variables that contribute to performance and injury risk such as sRPE, movement parameters, sleep quality, sweat rate, cardiac function, etc. In all cases, the implementation of these models using wearable sensor data stands to complement, rather than replace, the current decision-making and expertise of athletic trainers and team physicians.

## Author Contributions

DS, MT, EH, TG, JH, and DP contributed to the writing and editing of the manuscript. BG, CD, and JV contributed heavily to the editing of the manuscript. All authors contributed to the article and approved the submitted version.

## Conflict of Interest

JV serves as an educational consultant for Arthrex. TG works as a consultant to several high-performance organizations, including sporting teams, industry, military, and higher education institutions. The remaining authors declare that the research was conducted in the absence of any commercial or financial relationships that could be construed as a potential conflict of interest.

## Supplementary Material

Supplementary Figure 1 Workload-injury etiology model. Workloads affect injury etiology in three ways: (1) exposure (training and competition loads), (2) fitness (positive adaptations are associated with training which improve modifiable internal risk factors), and (3) fatigue (negative consequences associated with training temporarily causing decreased capacity in modifiable internal risk factors). Adapted and modified from Windt and Gabbett (2017).

## Abbreviations

ML: Machine Learning
RTP: Return to Play
GPS: Global Positioning System
RFID: Radio-Frequency Identification
HR: Heart Rate
HRV: Heart Rate Variability
SmO_2_: Muscle Oxygen Saturation
RR: Respiration Rate
sRPE: Session Rating of Perceived Exertion
ACWR: Acute to Chronic Workload Ratio
ACL: Anterior Cruciate Ligament
RFECV: Recursive Feature Elimination with Cross Validation
LASSO: Least Absolute Shrinkage and Selection Operator
HCM: Hypertrophic Cardiomyopathy
oHCM: Obstructive Hypertrophic Cardiomyopathy
AF: Atrial Fibrillation
ECG: Electrocardiogram
AHS: Apple Heart Study
KB: KardiaBand
TJS: Tommy John Surgery
COVID-19: Coronavirus Disease 2019
ACI: Acute Cardiac Injury.

## References

[B1] Aagaard P. Sharma S. McNamara David A. Joshi P. Ayers Colby R. de Lemos James A.. (2019). Arrhythmias and adaptations of the cardiac conduction system in former national football league players. J. Am. Heart Assoc. 8:e010401. 10.1161/JAHA.118.010401 31337251 PMC6761649

[B2] Andrade R. Wik E. H. Rebelo-Marques A. Blanch P. Whiteley R. Espregueira-Mendes J.. (2020). Is the acute: chronic workload ratio (ACWR) associated with risk of time-loss injury in professional team sports? a systematic review of methodology, variables and injury risk in practical situations. Sports Med. 50, 1613–1635. 10.1007/s40279-020-01308-6 32572824

[B3] Arazi H. Asadi A. Khalkhali F. Boullosa D. Hackney A. C. Granacher U.. (2020). Association between the acute to chronic workload ratio and injury occurrence in young male team soccer players: a preliminary study. Front. Physiol. 11:608. 10.3389/fphys.2020.00608 32670083 PMC7327085

[B4] Baggish A. L. Battle R. W. Beckerman J. G. Bove A. A. Lampert R. J. Levine B. D.. (2017). Sports cardiology: core curriculum for providing cardiovascular care to competitive athletes and highly active people. J. Am. Coll. Cardiol. 70, 1902–1918. 10.1016/j.jacc.2017.08.055 28982505

[B5] Bahr R. Krosshaug T. (2005). Understanding injury mechanisms: a key component of preventing injuries in sport. Br. J. Sports Med. 39, 324–329. 10.1136/bjsm.2005.018341 15911600 PMC1725226

[B6] Baker L. B. (2017). Sweating rate and sweat sodium concentration in athletes: a review of methodology and intra/interindividual variability. Sports Med. Auckl. NZ. 47, 111–128. 10.1007/s40279-017-0691-5 28332116 PMC5371639

[B7] Baker L. B. Barnes K. A. Anderson M. L. Passe D. H. Stofan J. R. (2016). Normative data for regional sweat sodium concentration and whole-body sweating rate in athletes. J. Sports Sci. 34, 358–368. 10.1080/02640414.2015.1055291 26070030

[B8] Baker L. B. Stofan J. R. Hamilton A. A. Horswill C. A. (2009). Comparison of regional patch collection vs. whole body washdown for measuring sweat sodium and potassium loss during exercise. J. Appl. Physiol. 107, 887–895. 10.1152/japplphysiol.00197.2009 19541738

[B9] Blanch P. Gabbett T. J. (2016). Has the athlete trained enough to return to play safely? The acute:chronic workload ratio permits clinicians to quantify a player's risk of subsequent injury. Br. J. Sports Med. 50, 471–475. 10.1136/bjsports-2015-095445 26701923

[B10] Bornn L. Ward P. Norman D. (2019). Training Schedule Confounds the Relationship Between Acute: Chronic Workload Ratio and Injury. Boston, MA: MIT Sloan Sports Anal Conf.

[B11] Bourdon P. C. Cardinale M. Murray A. Gastin P. Kellmann M. Varley M. C.. (2017). Monitoring athlete training loads: consensus statement. Int. J. Sports Physiol. Perform. 12, S2161–S2170. 10.1123/IJSPP.2017-0208 28463642

[B12] Bowen L. Gross A. S. Gimpel M. Bruce-Low S. Li F.-X. (2020). Spikes in acute:chronic workload ratio (ACWR) associated with a 5-7 times greater injury rate in english premier league football players: a comprehensive 3-year study. Br. J. Sports Med. 54, 731–738. 10.1136/bjsports-2018-099422 30792258 PMC7285788

[B13] Bowen L. Gross A. S. Gimpel M. Li F.-X. (2017). Accumulated workloads and the acute:chronic workload ratio relate to injury risk in elite youth football players. Br. J. Sports Med. 51, 452–459. 10.1136/bjsports-2015-095820 27450360 PMC5460663

[B14] Bumgarner J. M. Lambert C. T. Hussein A. A. Cantillon D. J. Baranowski B. Wolski K.. (2018). Smartwatch algorithm for automated detection of atrial fibrillation. J. Am. Coll. Cardiol. 71, 2381–2388. 10.1016/j.jacc.2018.03.003 29535065

[B15] Captur G. Heywood W. E. Coats C. Rosmini S. Patel V. Lopes L. R.. (2020). Identification of a multiplex biomarker panel for hypertrophic cardiomyopathy using quantitative proteomics and machine learning. Mol. Cell. Proteomics 19, 114–127. 10.1074/mcp.RA119.001586 31243064 PMC6944230

[B16] Carey D. L. Ong K. Whiteley R. Crossley K. M. Crow J. Morris M. E. (2018). Predictive modelling of training loads and injury in australian football. Int. J. Comput. Sci. Sport 17, 49–66. 10.2478/ijcss-2018-0002

[B17] Chambers R. Gabbett T. J. Cole M. H. Beard A. (2015). The use of wearable microsensors to quantify sport-specific movements. Sports Med. 45, 1065–1081. 10.1007/s40279-015-0332-9 25834998

[B18] Claudino J. G. de Oliveira Capanema D. de Souza T. V. Serrão J. C. Machado Pereira A. C. Nassis G. P. (2019). Current approaches to the use of artificial intelligence for injury risk assessment and performance prediction in team sports: a systematic review. Sports Med. Open 5:28. 10.1186/s40798-019-0202-3 31270636 PMC6609928

[B19] Colbert L. H. Matthews C. E. Havighurst T. C. Kim K. Schoeller D. A. (2011). Comparative validity of physical activity measures in older adults. Med. Sci. Sports Exerc. 43, 867–876. 10.1249/MSS.0b013e3181fc7162 20881882 PMC3303696

[B20] Colby M. J. Dawson B. Heasman J. Rogalski B. Gabbett T. J. (2014). Accelerometer and GPS-derived running loads and injury risk in elite australian footballers. J. Strength Cond. Res. 28, 2244–2252. 10.1519/JSC.0000000000000362 25054573

[B21] Count T. D. L. Jajack A. Heikenfeld J. Kasting G. B. (2019). Modeling glucose transport from systemic circulation to sweat. J. Pharm. Sci. 108, 364–371. 10.1016/j.xphs.2018.09.026 30273561

[B22] Crum E. M. O'Connor W. J. Van Loo L. Valckx M. Stannard S. R. (2017). Validity and reliability of the moxy oxygen monitor during incremental cycling exercise. Eur. J. Sport Sci. 17, 1037–43. 10.1080/17461391.2017.1330899 28557670

[B23] de Zambotti M. Claudatos S. Inkelis S. Colrain I. M. Baker F. C. (2015). Evaluation of a consumer fitness-tracking device to assess sleep in adults. Chronobiol. Int. 32, 1024–1028. 10.3109/07420528.2015.1054395 26158542 PMC4780439

[B24] DiFiori J. P. Benjamin H. J. Brenner J. S. Gregory A. Jayanthi N. Landry G. L.. (2014). Overuse injuries and burnout in youth sports: a position statement from the American medical society for sports medicine. Br. J. Sports Med. 48, 287–288. 10.1136/bjsports-2013-093299 24463910

[B25] Dodson C. C. Secrist E. S. Bhat S. B. Woods D. P. Deluca P. F. (2016). Anterior cruciate ligament injuries in national football league athletes from 2010 to 2013. Orthop. J. Sports Med. 4:2325967116631949 10.1177/2325967116631949 26998501 PMC4780097

[B26] Driggin E. Madhavan M. V. Bikdeli B. Chuich T. Laracy J. Bondi-Zoccai G.. (2020). Cardiovascular considerations for patients, health care workers, and health systems during the coronavirus disease 2019 (COVID-19) pandemic. J. Am. Coll. Cardiol. 75, 2352–2371. 10.1016/j.jacc.2020.03.031 32201335 PMC7198856

[B27] Dur O. Rhoades C. Ng M. S. Elsayed R. van Mourik R. Majmudar M. D. (2018). Design rationale and performance evaluation of the wavelet health wristband: benchtop validation of a wrist-worn physiological signal recorder. JMIR MHealth UHealth 6:e11040. 10.2196/11040 30327288 PMC6231731

[B28] Edwards S. White S. Humphreys S. Robergs R. O'Dwyer N. (2018). Caution using data from triaxial accelerometers housed in player tracking units during running. J. Sports Sci. 37, 810–818. 10.1080/02640414.2018.1527675 30306824

[B29] Ellis K. Godbole S. Kerr J. Lanckriet G. (2014). Multi-sensor physical activity recognition in free-living. Proc. ACM Int. Conf. Ubiquitous Comput. UbiComp Conf. 2014, 431–440. 10.1145/2638728.2641673 26247061 PMC4523301

[B30] Estes N. A. M. Madias C. (2017). Atrial fibrillation in athletes: a lesson in the virtue of moderation. JACC Clin. Electrophysiol. 3, 921–928. 10.1016/j.jacep.2017.03.019 29759716

[B31] Fidai M. S. Okoroha K. Meldau J. E. Borowsky P. A. Meta F. Lizzio V. A. (2018). Fatigue increases ACL injury risk in youth athletes: risk assessment study using drop-jump test. Orthop. J. Sports Med. 6:2325967118S00074. 10.1177/2325967118S00074

[B32] Fietze I. Strauch J. Holzhausen M. Glos M. Theobald C. Lehnkering H.. (2009). Sleep quality in professional ballet dancers. Chronobiol. Int. 26, 1249–1262. 10.1080/07420520903221319 19731116

[B33] Fox J. L. Scanlan A. T. Stanton R. Sargent C. (2020). Insufficient sleep in young athletes? Causes, consequences, and potential treatments. Sports Med. 50, 461–470. 10.1007/s40279-019-01220-8 31679145

[B34] Gabbett T. J. (2010). The development and application of an injury prediction model for noncontact, soft-tissue injuries in elite collision sport athletes. J. Strength Cond. Res. 24, 2593–2603. 10.1519/JSC.0b013e3181f19da4 20847703

[B35] Gabbett T. J. Nassis G. P. Oetter E. Pretorius J. Johnston N. Medina D.. (2017). The athlete monitoring cycle: a practical guide to interpreting and applying training monitoring data. Br. J. Sports Med. 51, 1451–2. 10.1136/bjsports-2016-097298 28646100

[B36] Gastin P. B. Mclean O. C. Breed R. V. P. Spittle M. (2014). Tackle and impact detection in elite Australian football using wearable microsensor technology. J. Sports Sci. 32, 947–953. 10.1080/02640414.2013.868920 24499311

[B37] Goh G. (2019). Game-Changing Analytics: How the Philadelphia 76ers Scored Big Wins Off the Court. Available online at: https://blog.datarobot.com/game-changing-analytics-how-the-philadelphia-76ers-scored-big-wins-off-the-court (accessed August 23, 2019).

[B38] Graham T. E. Pedersen P. K. Saltin B. (1987). Muscle and blood ammonia and lactate responses to prolonged exercise with hyperoxia. J. Appl. Physiol. 63, 1457–1462. 10.1152/jappl.1987.63.4.1457 3693180

[B39] Green E. M. van Mourik R. Wolfus C. Heitner S. B. Dur O. Semigran M. J. (2019). Machine learning detection of obstructive hypertrophic cardiomyopathy using a wearable biosensor. NPJ. Dig. Med. 2:57 10.1038/s41746-019-0130-0 PMC6591226 31304403

[B40] Harmon K. G. Suchsland M. Z. Prutkin J. M. Owens D. S. Aukerman D. F. Hwang C. E.. (2020). Comparison of cardiovascular screening in college athletes by history and physical examination with and without an electrocardiogram: efficacy and cost. Heart Rhythm. 17, 1649–1655. 10.1016/j.hrthm.2020.04.032 32380289

[B41] Hayes L. E. Boulos A. Cruz A. I. Jr. (2019). Risk factors for in-season injury in varsity collegiate cross-country athletes: an analysis of one season in 97 athletes. J. Sports Med. Phys. Fitness 59, 1536–1543. 10.23736/S0022-4707.19.09221-1 30758164

[B42] Hulin B. T. Gabbett T. J. Blanch P. Chapman P. Bailey D. Orchard J. W. (2014). Spikes in acute workload are associated with increased injury risk in elite cricket fast bowlers. Br. J. Sports Med. 48, 708–712. 10.1136/bjsports-2013-092524 23962877

[B43] Hulin B. T. Gabbett T. J. Caputi P. Lawson D. W. Sampson J. A. (2016). Low chronic workload and the acute:chronic workload ratio are more predictive of injury than between-match recovery time: a two-season prospective cohort study in elite rugby league players. Br. J. Sports Med. 50, 1008–1012. 10.1136/bjsports-2015-095364 26851288

[B44] Impellizzeri F. M. Wookcock S. Coutts A. J. Fanchini M. McCall A. Vigotsky A. D. (2020). Acute to random workload ratio is 'as' associated with injury as acute to actual chronic workload ratio: time to dismiss ACWR and its components. SportRxiv 10.31236/osf.io/e8kt4 33332011

[B45] Intille S. S. Lester J. Sallis J. F. Duncan G. (2012). New horizons in sensor development. Med. Sci. Sports Exerc. 44, S24–31. 10.1249/MSS.0b013e3182399c7d 22157771 PMC3245518

[B46] Ishøi L. Thorborg K. Kraemer O. Hölmich P. (2018). Return to sport and performance after hip arthroscopy for femoroacetabular impingement in 18- to 30-year-old athletes: a cross-sectional cohort study of 189 athletes. Am. J. Sports Med. 46, 2578–2587. 10.1177/0363546518789070 30067071

[B47] Jack R. A. Burn M. B. Sochacki K. R. McCulloch P. C. Lintner D. M. Harris J. D. (2018). Performance and return to sport after tommy john surgery among major league baseball position players. Am. J. Sports Med. 46, 1720–1726. 10.1177/0363546518762397 29601208

[B48] Jayanthi N. Kleithermes S. Dugas L. Pasulka J. Iqbal S. LaBella C. (2020). Risk of injuries associated with sport specialization and intense training patterns in young athletes: a longitudinal clinical case-control study. Orthop. J. Sports Med. 8:232596712092276. 10.1177/2325967120922764 32637428 PMC7318830

[B49] Jayanthi N. Pinkham C. Dugas L. Patrick B. Labella C. (2013). Sports specialization in young athletes: evidence-based recommendations. Sports Health. 5, 251–257. 10.1177/1941738112464626 24427397 PMC3658407

[B50] Jayanthi N. A. LaBella C. R. Fischer D. Pasulka J. Dugas L. R. (2015). Sports-specialized intensive training and the risk of injury in young athletes: a clinical case-control study. Am. J. Sports Med. 43, 794–801. 10.1177/0363546514567298 25646361

[B51] Jia W. Bandodkar A. J. Valdés-Ramírez G. Windmiller J. R. Yang Z. Ramírez J.. (2013). Electrochemical tattoo biosensors for real-time noninvasive lactate monitoring in human perspiration. Anal. Chem. 85, 6553–6560. 10.1021/ac401573r 23815621

[B52] Juliff L. E. Halson S. L. Peiffer J. J. (2015). Understanding sleep disturbance in athletes prior to important competitions. J. Sci. Med. Sport 18, 13–18. 10.1016/j.jsams.2014.02.007 24629327

[B53] Katz A. Sahlin K. (1988). Regulation of lactic acid production during exercise. J. Appl. Physiol. 65, 509–518. 10.1152/jappl.1988.65.2.509 3049511

[B54] LaPrade R. F. Agel J. Baker J. Brenner J. S. Cordasco F. A. Côté J.. (2016). AOSSM early sport specialization consensus statement. Orthop. J. Sports Med. 4:2325967116644241. 10.1177/2325967116644241 27169132 PMC4853833

[B55] Li R. T. Salata M. J. Rambhia S. Sheehan J. Voos J. E. (2020). Does overexertion correlate with increased injury? The relationship between player workload and soft tissue injury in professional American football players using wearable technology. Sports Health 12, 66–73. 10.1177/1941738119868477 31469616 PMC6931176

[B56] Losciale J. M. Zdeb R. M. Ledbetter L. Reiman M. P. Sell T. C. (2018). The association between passing return-to-sport criteria and second anterior cruciate ligament injury risk: a systematic review with meta-analysis. J. Orthop. Sports Phys. Ther. 49, 43–54. 10.2519/jospt.2019.8190 30501385

[B57] Malhotra A. Dhutia H. Finocchiaro G. Gati S. Beasley I. Clift P. (2018). Outcomes of cardiac screening in adolescent soccer players. N. Engl. J. Med. 379, 524–534. 10.1056/NEJMoa1714719 30089062

[B58] Maron Barry J. Friedman Richard A. Kligfield P. Levine Benjamin D. Viskin S. Chaitman Bernard R.. (2014). Assessment of the 12-lead ECG as a screening test for detection of cardiovascular disease in healthy general populations of young people (12-25 years of age). circulation. Am. Heart Assoc. 130, 1303–34. 10.1161/CIR.0000000000000025 25223981

[B59] Maron M. S. Hellawell J. L. Lucove J. C. Farzaneh-Far R. Olivotto I. (2016). Occurrence of clinically diagnosed hypertrophic cardiomyopathy in the united states. Am. J. Cardiol. 117, 1651–1654. 10.1016/j.amjcard.2016.02.044 27006153

[B60] Meeuwisse W. H. (1994). Assessing causation in sport injury: a multifactorial model. Clin. J. Sport Med. 4, 166–170. 10.1097/00042752-199407000-00004

[B61] Meeuwisse W. H. Tyreman H. Hagel B. Emery C. (2007). A dynamic model of etiology in sport injury: the recursive nature of risk and causation. Clin. J. Sport Med. 17, 215–219. 10.1097/JSM.0b013e3180592a48 17513916

[B62] Mishra T. Wang M. Metwally A. A. Bogu G. K. Brooks A. W. Bahmani A.. (2020). Pre-symptomatic detection of COVID-19 from smartwatch data. Nat. Biomed. Eng. 4, 1–13. 10.1038/s41551-020-00640-6 33208926 PMC9020268

[B63] Moxy Monitor (2020). Muscle Oxygen Monitor. Moxy Monitor. Available online at: https://www.moxymonitor.com/ (accessed July 3, 2020).

[B64] Mueller L. M. Bloomer B. A. Durall C. J. (2014). Which outcome measures should be used to determine readiness to play after ACL reconstruction? J. Sport Rehabil. 23, 158–164. 10.1123/JSR.2013-0018 23981536

[B65] Murray N. B. Gabbett T. J. Townshend A. D. Blanch P. (2017). Calculating acute:chronic workload ratios using exponentially weighted moving averages provides a more sensitive indicator of injury likelihood than rolling averages. Br. J. Sports Med. 51, 749–754. 10.1136/bjsports-2016-097152 28003238

[B66] Musahl V. Karlsson J. (2019). Anterior cruciate ligament tear. N. Engl. J. Med. 380, 2341–2348. 10.1056/NEJMcp1805931 31189037

[B67] Musahl V. Karlsson J. Krutsch W. Mandelbaum B. R. Espregueira-Mendes J. d'Hooghe P. (2018). Return to Play in Football: An Evidence-based Approach. Berlin Heidelberg: Springer-Verlag 10.1007/978-3-662-55713-6

[B68] Myer G. D. Faigenbaum A. D. Cherny C. E. Heidt R. S. Hewett T. E. (2011). Did the NFL lockout expose the achilles heel of competitive sports? J. Orthop. Sports Phys. Ther. 41, 702–5. 10.2519/jospt.2011.0107 21941038

[B69] Myers N. L. Mexicano G. Aguilar K. V. (2020). The association between noncontact injuries and the acute-chronic workload ratio in elite-level athletes: a critically appraised topic. J. Sport Rehabil. 29, 1–13. 10.1123/jsr.2018-0207 31094616

[B70] Nazari G. Bobos P. MacDermid J. C. Sinden K. E. Richardson J. Tang A. (2018). Psychometric properties of the zephyr bioharness device: a systematic review. BMC Sports Sci. Med. Rehabil. 10:6. 10.1186/s13102-018-0094-4 29484191 PMC5822593

[B71] O'Connor M. Minkara A. A. Westermann R. W. Rosneck J. Lynch T. S. (2018). Return to play after hip arthroscopy: a systematic review and meta-analysis. Am. J. Sports Med. 46, 2780–2788. 10.1177/0363546518759731 29595996

[B72] Paterno M. V. Huang B. Thomas S. Hewett T. E. Schmitt L. C. (2017). Clinical factors that predict a second ACL injury after ACL reconstruction and return to sport: preliminary development of a clinical decision algorithm. Orthop. J. Sports Med. 5:2325967117745279. 10.1177/2325967117745279 29318172 PMC5753959

[B73] Peake J. M. Kerr G. Sullivan J. P. (2018). A critical review of consumer wearables, mobile applications, and equipment for providing biofeedback, monitoring stress, and sleep in physically active populations. Front. Physiol. 9:743. 10.3389/fphys.2018.00743 30002629 PMC6031746

[B74] Phelan D. Kim J. H. Chung E. H. (2020). A game plan for the resumption of sport and exercise after coronavirus disease 2019 (COVID-19) infection. JAMA Cardiol. 5, 1085–1086. 10.1001/jamacardio.2020.2136 32402054

[B75] Rasmusen H. Schmied C. (2020). Medical Evaluation of Athletes: Medical History and Physical Examination (Springer), 95–111. 10.1007/978-3-030-35374-2_6

[B76] Rossi A. Pappalardo L. Cintia P. Iaia F. M. Fernàndez J. Medina D. (2018). Effective injury forecasting in soccer with GPS training data and machine learning. PLoS ONE 13:e0201264. 10.1371/journal.pone.0201264 30044858 PMC6059460

[B77] Ryan J. L. Pracht E. E. Orban B. L. (2019). Inpatient and emergency department costs from sports injuries among youth aged 5-18 years. BMJ Open Sport Exerc. Med. 5:e000491. 10.1136/bmjsem-2018-000491 31191961 PMC6539161

[B78] Sampson J. A. Murray A. Williams S. Halseth T. Hanisch J. Golden G.. (2018). Injury risk-workload associations in NCAA American college football. J. Sci. Med. Sport 21, 1215–1220. 10.1016/j.jsams.2018.05.019 29843960

[B79] Sargent C. Lastella M. Halson S. Roach G. (2014). The impact of training schedules on the sleep and fatigue of elite athletes. Chronobiol. Int. 31, 1160–1168. 10.3109/07420528.2014.957306 25222347

[B80] Schwellnus M. Soligard T. Alonso J.-M. Bahr R. Clarsen B. Dijkstra H. P.. (2016). How much is too much? (Part 2) international olympic committee consensus statement on load in sport and risk of illness. Br. J. Sports Med. 50, 1043–1052. 10.1136/bjsports-2016-096572 27535991 PMC5013087

[B81] Scott H. Lack L. Lovato N. (2020). A systematic review of the accuracy of sleep wearable devices for estimating sleep onset. Sleep Med. Rev. 49:101227. 10.1016/j.smrv.2019.101227 31901524

[B82] Secrist E. S. Bhat S. B. Dodson C. C. (2016). The financial and professional impact of anterior cruciate ligament injuries in national football league athletes. Orthop. J. Sports Med. 4:2325967116663921. 10.1177/2325967116663921 27631017 PMC5010185

[B83] Sekiguchi Y. Adams W. M. Benjamin C. L. Curtis R. M. Giersch G. E. W. Casa D. J. (2019). Relationships between resting heart rate, heart rate variability and sleep characteristics among female collegiate cross-country athletes. J. Sleep Res. 28:e12836. 10.1111/jsr.12836 30843295

[B84] Seow D. Graham I. Massey A. (2020). Prediction models for musculoskeletal injuries in professional sporting activities: a systematic review. Transl. Sports Med. 3, 505–517. 10.1002/tsm2.181

[B85] Seshadri Dhruv R. Barbara B. Dalton B. Penny H. Drummond Colin K. Desai Milind Y.. (2020). Accuracy of apple watch for detection of atrial fibrillation. Circulation 141, 702–3. 10.1161/CIRCULATIONAHA.119.044126 32091929

[B86] Seshadri D. R. Davies E. V. Harlow E. R. Hsu J. J. Knighton S. C. Walker T. A. (2020). Wearable sensors for COVID-19: a call to action to harness our digital infrastructure for remote patient monitoring and virtual assessments. Front. Dig. Health 2:8 10.3389/fdgth.2020.00008 PMC8521919 34713021

[B87] Seshadri D. R. Drummond C. Craker J. Rowbottom J. R. Voos J. E. (2017). Wearable devices for sports: new integrated technologies allow coaches, physicians, and trainers to better understand the physical demands of athletes in real time. IEEE Pulse 8, 38–43. 10.1109/MPUL.2016.2627240 28129141

[B88] Seshadri D. R. Li R. T. Voos J. E. Rowbottom J. R. Alfes C. M. Zorman C. A. (2019b). Wearable sensors for monitoring the physiological and biochemical profile of the athlete. NPJ. Dig. Med. 2:72 10.1038/s41746-019-0150-9 PMC6646404 31341957

[B89] Seshadri D. R. Li R. T. Voos J. E. Rowbottom J. R. Alfes C. M. Zorman C. A. (2019c). Wearable sensors for monitoring the internal and external workload of the athlete. NPJ Digit Med. 2:71 10.1038/s41746-019-0149-2 31372506 PMC6662809

[B90] Seshadri D. R. Magliato S. Voos J. E. Drummond C. (2019a). Clinical translation of biomedical sensors for sports medicine. J. Med. Eng. Technol. 43, 66–81. 10.1080/03091902.2019.1612474 31119965

[B91] Shrier I. (2015). Strategic assessment of risk and risk tolerance (StARRT) framework for return-to-play decision-making. Br. J. Sports Med. 49, 1311–1315. 10.1136/bjsports-2014-094569 26036678

[B92] Shrier I. Matheson G. O. Boudier-Revéret M. Steele R. J. (2015). Validating the three-step return-to-play decision model. Scand. J. Med. Sci. Sports 25, e231–239. 10.1111/sms.12306 25098497

[B93] Simpson N. S. Gibbs E. L. Matheson G. O. (2017). Optimizing sleep to maximize performance: implications and recommendations for elite athletes. Scand. J. Med. Sci. Sports 27, 266–274. 10.1111/sms.12703 27367265

[B94] Soligard T. Schwellnus M. Alonso J.-M. Bahr R. Clarsen B. Dijkstra H. P.. (2016). How much is too much? (Part 1) international olympic committee consensus statement on load in sport and risk of injury. Br. J. Sports Med. 50, 1030–1041. 10.1136/bjsports-2016-096581 27535989

[B95] Stern B. D. Hegedus E. J. Lai Y.-C. (2020). Injury prediction as a non-linear system. Phys. Ther. Sport 41, 43–48. 10.1016/j.ptsp.2019.10.010 31733565

[B96] touchCARDIO (2020). Electrocardiography in Athletes - How to Identify High-risk Subjects? Available online at: https://www.touchcardio.com/electrocardiography-in-athletes-how-to-identify-high-risk-subjects/ (accessed July 2, 2020).

[B97] Turagam M. K. Flaker G. C. Velagapudi P. Vadali S. Alpert M. A. (2015). Atrial fibrillation in athletes: pathophysiology, clinical presentation, evaluation and management. J. Atr. Fibrillation 8:1309. 10.4022/jafib.1309 27957228 PMC5135187

[B98] Turakhia M. P. Desai M. Hedlin H. Rajmane A. Talati N. Ferris T.. (2018). Rationale and design of a large-scale, app-based study to identify cardiac arrhythmias using a smartwatch: the apple heart study. Am. Heart J. 207, 66–75. 10.1016/j.ahj.2018.09.002 30392584 PMC8099048

[B99] Vamathevan J. Clark D. Czodrowski P. Dunham I. Ferran E. Lee G.. (2019). Applications of machine learning in drug discovery and development. Nat. Rev. Drug Discov. 18, 463–477. 10.1038/s41573-019-0024-5 30976107 PMC6552674

[B100] Van de Water A. T. M. Holmes A. Hurley D. A. (2011). Objective measurements of sleep for non-laboratory settings as alternatives to polysomnography - a systematic review. J. Sleep Res. 20, 183–200. 10.1111/j.1365-2869.2009.00814.x 20374444

[B101] von Rosen P. Frohm A. Kottorp A. Fridén C. Heijne A. (2017). Multiple factors explain injury risk in adolescent elite athletes: applying a biopsychosocial perspective. Scand. J. Med. Sci. Sports 27, 2059–2069. 10.1111/sms.12855 28207969

[B102] Webster K. E. Hewett T. E. (2018). Meta-analysis of meta-analyses of anterior cruciate ligament injury reduction training programs. J. Orthop. Res. 36, 2696–2708. 10.1002/jor.24043 29737024

[B103] Welch W. A. Bassett D. R. Thompson D. L. Freedson P. S. Staudenmayer J. W. John D.. (2013). Classification accuracy of the wrist-worn gravity estimator of normal everyday activity accelerometer. Med. Sci. Sports Exerc. 45, 2012–2019. 10.1249/MSS.0b013e3182965249 23584403 PMC3778030

[B104] Wellman A. D. Coad S. C. Goulet G. C. McLellan C. P. (2016). Quantification of competitive game demands of NCAA division i college football players using global positioning systems. J. Strength Cond. Res. 30, 11–19. 10.1519/JSC.0000000000001206 26382134

[B105] Whiteley R. Massey A. Gabbett T. Blanch P. Cameron M. Conlan G. (2020). Match high-speed running distances are often suppressed following return from hamstring strain injury in professional footballers. Sports Health 12:1941738120964456 10.1177/1941738120964456 PMC8079800 33151808

[B106] Wiggins A. J. Grandhi R. K. Schneider D. K. Stanfield D. Webster K. E. Myer G. D. (2016). Risk of secondary injury in younger athletes after anterior cruciate ligament reconstruction. Am. J. Sports Med. 44, 1861–1876. 10.1177/0363546515621554 26772611 PMC5501245

[B107] Windt J. Gabbett T. J. (2017). How do training and competition workloads relate to injury? The workload-injury aetiology model. Br. J. Sports Med. 51, 428–435. 10.1136/bjsports-2016-096040 27418321

[B108] Wundersitz D. W. T. Josman C. Gupta R. Netto K. J. Gastin P. B. Robertson S. (2015). Classification of team sport activities using a single wearable tracking device. J. Biomech. 48, 3975–3981. 10.1016/j.jbiomech.2015.09.015 26472301

[B109] Yokus M. A. Songkakul T. Pozdin V. A. Bozkurt A. Daniele M. A. (2020). Wearable multiplexed biosensor system toward continuous monitoring of metabolites. Biosens Bioelectron. 153:112038. 10.1016/j.bios.2020.112038 31989942

[B110] Zaffagnini S. Grassi A. Serra M. Marcacci M. (2015). Return to sport after ACL reconstruction: how, when and why? A narrative review of current evidence. Joints 3, 25–30. 10.11138/jts/2015.3.1.025 26151036 PMC4469040

[B111] Zebra The NFL (2019). Official on-field player-tracking. Zebra. Zebra Technol. Available online at: https://www.zebra.com/us/en/nfl.html (accessed August 22, 2019).

