# Supplementary figures and images for: Wearable Technology and Analytics as a Complementary Toolkit to Optimize Workload and to Reduce Injury Burden
Source: Front Sports Act Living. 2021 Jan 21;2:630576. doi: 10.3389/fspor.2020.630576 (PMC7859639; doi:10.3389/fspor.2020.630576)

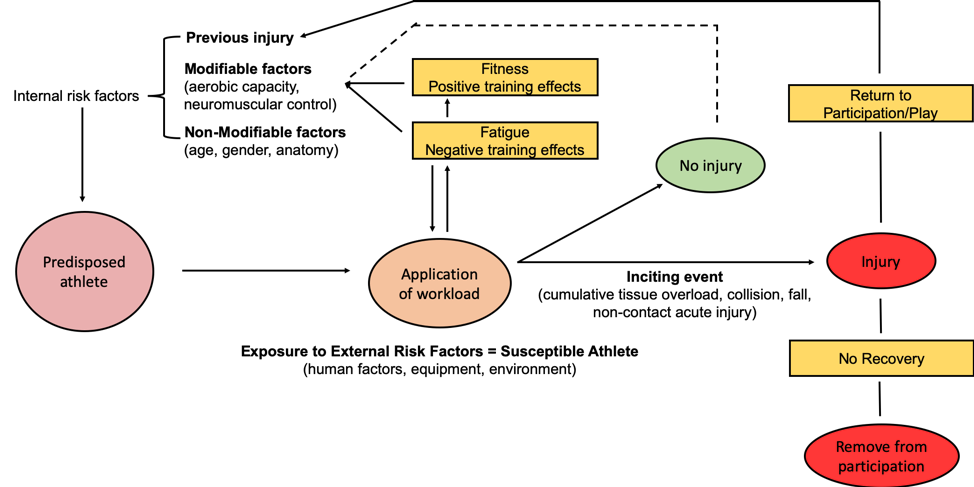

Supplement: Supplementary Figure 1 — Workload-injury etiology model. Workloads affect injury etiology in three ways: (1) exposure (training and competition loads), (2) fitness (positive adaptations are associated with training which improve modifiable internal risk factors), and (3) fatigue (negative consequences associated with training temporarily causing decreased capacity in modifiable internal risk factors). Adapted and modified from Windt and Gabbett (2017). [file Image_1.PNG]
